# Opposing seasonal trends in source water and sugar dampen intra‐annual variability in tree rings oxygen isotopes

**DOI:** 10.1111/nph.70223

**Published:** 2025-05-20

**Authors:** Paul Szejner, Yu Tang, Charlotte Angove, Pauliina Schiestl‐Aalto, Elina Sahlstedt, Giles Young, Daniel B. Nelson, Ansgar Kahmen, Matthias Saurer, Katja T. Rinne‐Garmston

**Affiliations:** ^1^ Stable Isotope Laboratory of Luke (SILL) Natural Resources Institute Finland (Luke) Latokartanonkaari 9 FI‐00790 Helsinki Finland; ^2^ College of Urban and Environmental Sciences Peking University Beijing 100871 China; ^3^ Institute for Atmospheric and Earth System Research (INAR)/Physics, Faculty of Science University of Helsinki PO Box 68 FI‐00014 Helsinki Finland; ^4^ Department of Environmental Sciences – Botany University of Basel 4056 Basel Switzerland; ^5^ Forest Dynamics Swiss Federal Institute for Forest, Snow and Landscape Research WSL Zürcherstrasse 111 8903 Birmensdorf Switzerland

**Keywords:** boreal forests, high‐resolution dendrochronology, needle sugars, phloem sugars, post‐photosynthetic fractionation, proxy records, stable isotopes

## Abstract

- Variations of oxygen isotopes δ^18^O in tree rings provide critical insights into past climate and tree physiological processes, yet the mechanisms shaping the intra‐annual δ^18^O signals remain incompletely understood. To address this gap, we investigated how seasonal changes in source water, leaf water, and sugars influence δ^18^O recorded along the tree rings of *Pinus sylvestris* in Finland.
- We conducted a seasonal analysis measuring δ^18^O from needle water, source water, and phloem sugars and investigated the fraction of oxygen isotope exchange during wood formation.
- We found that seasonal δ^18^O amplitudes are significantly reduced from leaf water to tree rings, driven by opposing seasonal patterns in increasing source water δ^18^O and decreasing evaporative enrichment as relative humidity increases. Additionally, the isotope exchange between source water and phloem sugars further dampens seasonal δ^18^O signals in the rings.
- Our findings show that oxygen isotope exchange is critical in shaping δ^18^O signals, influencing the role of source water and relative humidity recorded on intra‐annual resolution. This refined understanding helps interpret tree physiological responses under changing conditions and improves climate reconstructions based on tree rings using intra‐annual resolution.

## Introduction

Understanding the interactions between tree physiology and environmental factors is crucial for assessing the impact of climate change on forest ecosystems. Tree rings serve as valuable proxy archives that integrate physiological responses throughout the growing season, reflecting adjustments to seasonal and environmental changes (Monson *et al*., 2018; Szejner *et al*., 2021). By integrating these seasonal signals, tree rings not only serve as archives of past climate variability but also improve the precision of climate reconstructions, thereby informing predictive models of forest response (Rinne *et al*., 2013; Esper *et al*., 2017; Belmecheri *et al*., 2018; Treydte *et al*., 2023).

The oxygen isotope composition (δ^18^O) of water and organic matter in trees offers insights into the processes governing tree water use and carbon allocation dynamics. These processes are influenced by environmental factors such as relative humidity (RH) and play a significant role in forest responses to climate change at seasonal and interannual scales (Barbour *et al*., 2001; Gessler *et al*., 2013; Ireson *et al*., 2015; Ruiz‐Pérez & Vico, 2020). The δ^18^O signal in tree rings results from the interplay between climatic variations and the plant's physiological responses, making it a powerful tool for reconstructing past environmental conditions (Roden *et al*., 2000; Barbour *et al*., 2002; Leavitt, 2002).

At the leaf level, the δ^18^O of leaf water is influenced by the plant source water δ^18^O value, transpiration processes, and environmental factors such as RH. During transpiration, heavier water isotopologues like H_2_^18^O are preferentially left behind, leading to isotopically enriched leaf water compared to the xylem source water (Craig & Gordon, 1965; Cernusak *et al*., 2018). Leaf water δ^18^O is strongly correlated with atmospheric RH; arid conditions result in more ^18^O‐enriched leaf water compared to humid environments (Roden *et al*., 2000; Cernusak *et al*., 2022). However, the basic Craig & Gordon model does not account for mixing between ^18^O‐enriched leaf water and new source water supplied to the leaf, which can affect the δ^18^O value of synthesized sugars (Roden *et al*., 2015; Fiorella *et al*., 2022). Such effects can be incorporated using the Peclet effect or mixing model modifications (Barbour & Farquhar, 2000; Cernusak *et al*., 2016; Kannenberg *et al*., 2021).

The δ^18^O of sugars synthesized during photosynthesis carries the isotopic signature of leaf water, allowing us to track carbon allocation as these sugars move from leaves to stems and other plant organs (Gessler *et al*., 2009). After photosynthesis, sugars are transported from the leaves to sites of wood formation. During cellulose synthesis, isotopic exchange can occur between the oxygen atoms in carbohydrates and the surrounding available water. This exchange is characterized in modeling by the term *p*_x_ × *p*_ex_ (Sternberg *et al*., 1986; Barbour & Farquhar, 2000) where *p*_ex_ is the fraction of exchanged oxygen atoms and *p*_x_ is defined as the fraction of (^18^O unenriched) source water vs ^18^O‐enriched leaf water at the cellulose synthesis site. Here, we focus on stem wood formation, and thus, we assume *p*_x_ to be 1 because we should not expect enriched water present during the wood synthesis. Therefore, we do not use the term *p*_x_ further in the equations. However, understanding *p*_ex_ is essential for accurately interpreting the δ^18^O signal in tree ring cellulose, as it reflects the mixing effect between the δ^18^O of sucrose and that of source water at the site of wood formation (Hill *et al*., 1995; Song *et al*., 2014).

Despite numerous studies demonstrating the capability of tree‐ring δ^18^O as a climate proxy, there remains a gap in our understanding of the processes affecting δ^18^O during tree ring formation and how these processes reflect past environmental conditions (Gessler *et al*., 2014). In particular, the temporal integration (Leppä *et al*., 2022; Martínez‐Sancho *et al*., 2023) and influence of mixing factors – such as leaf‐level evaporative enrichment, variation in source water, and oxygen exchange during cellulose or wood synthesis, *p*_ex_ – are not yet fully understood. Recent work has shown that *p*_ex_ can be influenced by the turnover rates of nonstructural carbohydrates (Song *et al*., 2014), rising atmospheric CO_2_ (Morgner *et al*., 2024), and RH or aridity (Cheesman & Cernusak, 2017; Holloway‐Phillips *et al*., 2023; Martínez‐Sancho *et al*., 2023; Bailey *et al*., 2025). Studying the δ^18^O of sugars in a high temporal resolution provides a more detailed perspective on these dynamics by capturing the isotopic signal from leaf water as it is transported to the stem (Fiorella *et al*., 2022). This approach clarifies whether tree ring δ^18^O reflects newly assimilated carbon or stored reserves and how these pools integrate environmental changes, enhancing our ability to interpret δ^18^O‐based proxies.

In this study, we investigate the seasonal δ^18^O variations in intra‐annual tree rings of Scots pine (*Pinus sylvestris* L.) at the study sites Hyytiälä (HYY) and Värriö (VAR) in Finland during the growing seasons of 2018 and 2019. Focusing on the mechanistic progression from source water to photosynthates (e.g. sugars) and finally to the δ^18^O variations fixed in wood (Offermann *et al*., 2011; Gessler *et al*., 2013, 2014), we aim to: quantify the δ^18^O offsets between water, sugars, and wood as carbon and water pools at an intra‐annual resolution (using multiple subsections per ring); examine how seasonal variations in δ^18^O are integrated into the different pools; and analyze how different temporal integration periods affect the climate signals from intra‐annual tree‐ring δ^18^O, examining temporal correlations.

## Materials and Methods

### Study sites

The two boreal forests studied were the Hyytiälä Forestry Field Station (HYY; 61.85°N, 24.29°E) and the Värriö Subarctic Research Station (VAR; 67.75°N, 29.61°E), both dominated by Scots pine (*Pinus sylvestris* L.). HYY, located in southern Finland at 180 m above sea level (m asl), is a 55‐yr‐old managed forest with a dense structure (1304 trees ha^−1^, average height 20 m) (Kolari *et al*., 2022). By contrast, VAR, at 395 m asl near the arctic‐alpine tree line, is a natural, less dense forest (750 trees ha^−1^) with shorter trees averaging 10 m, mostly aged 60–70 yr (Kolari *et al*., 2023).

During the 2018 and 2019 growing seasons, we systematically collected samples from both HYY and VAR. These included three main pools: water samples from various sources (rain, soil, twig, and needle water); water‐soluble carbohydrates (WSCs) from different tree components (twigs, phloem, current‐year needles (N0), 1‐yr‐old needles (N1), and roots); and wood samples from tree rings.

Sampling commenced in May and continued until October, aligning with the radial growth phases (Supporting Information Figs 1, S1–S7). This sampling approach leverages the established synchrony between radial growth and carbon uptake by leaves – that is, carbohydrates produced during the growing season are used for radial growth (Rinne‐Garmston *et al*., 2023) with a time lag of 1–5 d (Tang *et al*., 2023).

**Fig. 1:**
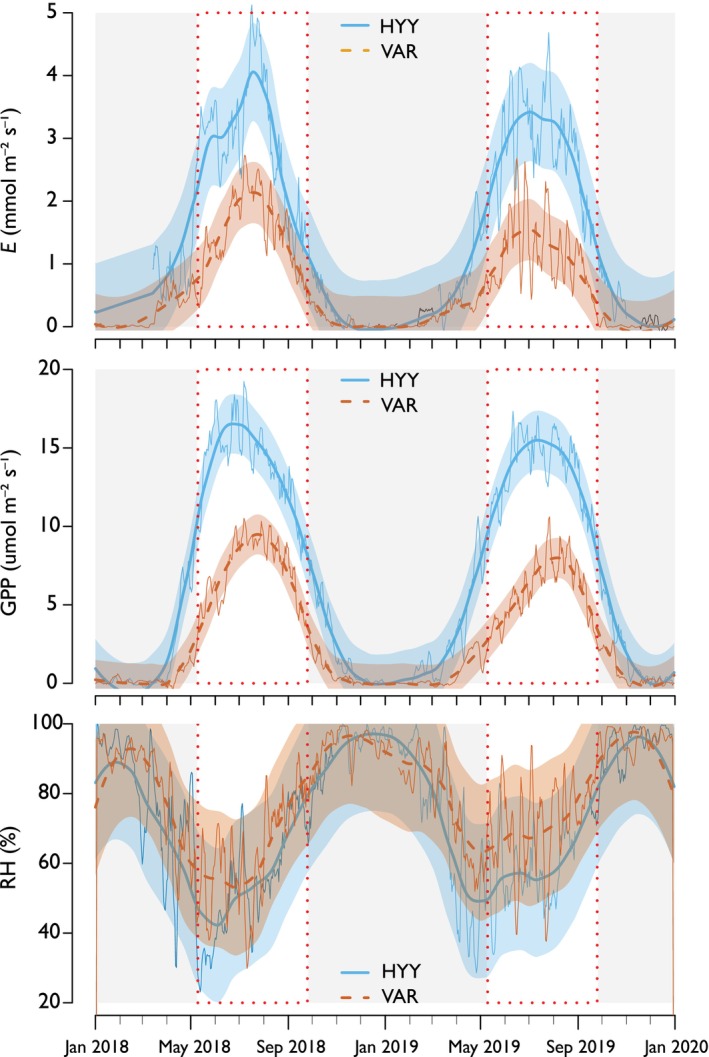
Environmental variables and instrumental records for 2018 and 2019 at Hyytiälä (HYY) and Värriö (VAR) sites. The data are derived from eddy covariance micrometeorological stations at the study sites, providing detailed insights into local environmental conditions. A 15‐d running integration, starting from the hourly dataset, was utilized, and the data can be accessed at https://smear.avaa.csc.fi/download. The integration for evapotranspiration (*E*, mmol m^−2^ s^−1^) and gross primary productivity (GPP, μmol m^−2^ s^−1^) was performed using the summation of GPP. The relative humidity (RH) was calculated using the mean values of the daily average within a daytime window from 9:00 h to 15:00 h for all the integrations. Gray boxes represent periods when trees were not monitored for this study, while the white background indicates the time frame when measurements were conducted and when trees were active in secondary growth (transparent envelopes show a 95% CI).

### Source and leaf water sampling

Cumulative monthly rainwater was collected throughout the year at each site using an evaporation‐free collector, as described by Gröning *et al*. (2012). For soil water analysis, we collected soil samples biweekly to monthly from random locations near the sampled trees, six times per growing season. At HYY, soil samples were taken from depths of 2, 10, and 18 cm, while at VAR, the sampling was limited to a depth of 10 cm. All soil samples were individually sealed in 12‐ml exetainer vials (Labco, Lampeter, UK).

We collected N0 and N1 needles with their corresponding twigs (without bark) from the sunlit branches of five mature trees. This collection was carried out during the window from 13:00 h to 16:00 h for logistical considerations and to systematically collect the data, which minimizes uncertainties caused by diurnal variations in isotopic composition if collected at different time windows (Tang *et al*., 2023). At HYY, samples were collected at an elevation of 18 m using a walk‐in scaffolding tower. At VAR, samples were accessed at 10 m using tree‐attached ladders. Samples were immediately placed in a cool box and stored at −20°C until isotopic analysis. This sampling process was repeated 6–9 times throughout each growing season, ensuring robust temporal representation.

### WSC sampling

For phloem WSCs, we utilized a 2‐cm‐diameter metal puncher to collect phloem samples from the main stem at 1.3 m height from five mature trees at each site. This procedure was carried out six times per season and site. We also collected WSCs from needles and twigs, differentiating between current‐year and 1‐yr‐old samples, ensuring they were taken from sunlit positions on the five sampled trees. Sampling was conducted weekly, with 20 to 23 collections per year and site (Figs S1–S7), all between 13:00 h and 16:00 h.

### Intra‐annual tree ring analysis

Samples were collected for tree ring analysis after the cessation of growth in 2019. At HYY, five mature trees were felled, and disks were collected at *c*. 1.3 m height. At VAR, one 5‐mm tree core was collected at breast height (*c*. 1.3 m) from five mature trees. All sampled trees at each site were of similar size and had comparable growth conditions. Trees sampled for tree rings were not the same as those for needles and phloem to minimize damage to the trees under long‐term monitoring (see Tables S1, S2). Despite this, the average δ^18^O values and trends from five trees should still accurately represent the study sites (Leavitt & Long, 1984; McCarroll & Loader, 2004; Siegwolf *et al*., 2022).

### Sample treatment and isotope analysis

#### Water samples

Water was extracted from soil, twigs, and needles using cryogenic vacuum distillation, following established techniques (West *et al*., 2006; Diao *et al*., 2022), performed at the Swiss Federal Institute for Forest, Snow and Landscape Research (WSL). Monthly precipitation samples were collected in HYY and VAR, and subsets were pipetted into 2‐ml vials for isotope analysis. Precipitation, plant, and soil‐extracted water from HYY and VAR were analyzed in three laboratories by thermal conversion elemental analyzer‐isotope ratio mass spectrometry (TCEA‐IRMS) to determine the δ^18^O values of these samples, of which the data from HYY have been previously published in Leppä *et al*. (2022). Precipitation samples from VAR (June 2018–May 2019) were analyzed at the Stable Isotope Laboratory in Luke (SILL) and (June 2019–August 2019) at the Stable Isotope Laboratory of the University of Basel, Switzerland. Plant and soil‐extracted water samples from 2018 at VAR were analyzed for δ^18^O at the Stable Isotope Research Laboratory of WSL (Birmensdorf, Switzerland), while corresponding samples from 2019 were analyzed at the Stable Isotope Laboratory of the University of Basel, Switzerland. For all samples, measurement precision was 0.3‰ or better based on replicate measurements and concurrent analysis of quality control waters.

#### WSC samples

A total of 434 WSC samples (Tables S1–S4) were extracted and purified following established methods (Wanek *et al*., 2001; Rinne *et al*., 2012; Lehmann *et al*., 2020). Approximately 60 mg of powder sample was mixed with 1.5 ml of deionized water in 2‐ml reaction vials, vortexed, and then, subjected to an 85°C water bath for 30 min. Post‐heating, the vials were centrifuged at 10 000 ***g*** for 2 min. The clear supernatant obtained was purified using Dionex OnGuard II H, A & P cartridges (Thermo Fisher Scientific) to remove interfering substances, such as amino acids, organic acids, and phenolic compounds. The purified samples were then stored at −20°C until isotope analysis. Isotope analysis of WSCs from HYY (Leppä *et al*., 2022) and VAR (this study) was performed at SILL. For the analysis, aliquots of solubilized WSC were pipetted into silver capsules (IVA Analysentechik, Meerbusch, Germany) and freeze‐dried. The sealed capsules were subsequently analyzed using a thermal conversion elemental analyzer (TCEA) connected to an isotope ratio mass spectrometer (IRMS). The measurements were calibrated against IAEA‐601 (23.14‰) and two in‐house reference materials, sucrose and lactose (36.6‰ and 21.1‰, respectively, from Sigma‐Aldrich). Measurement precision was 0.2‰, determined from repeated measurements of a quality control (QC) material.

#### Wood samples

**Fig. 2:**
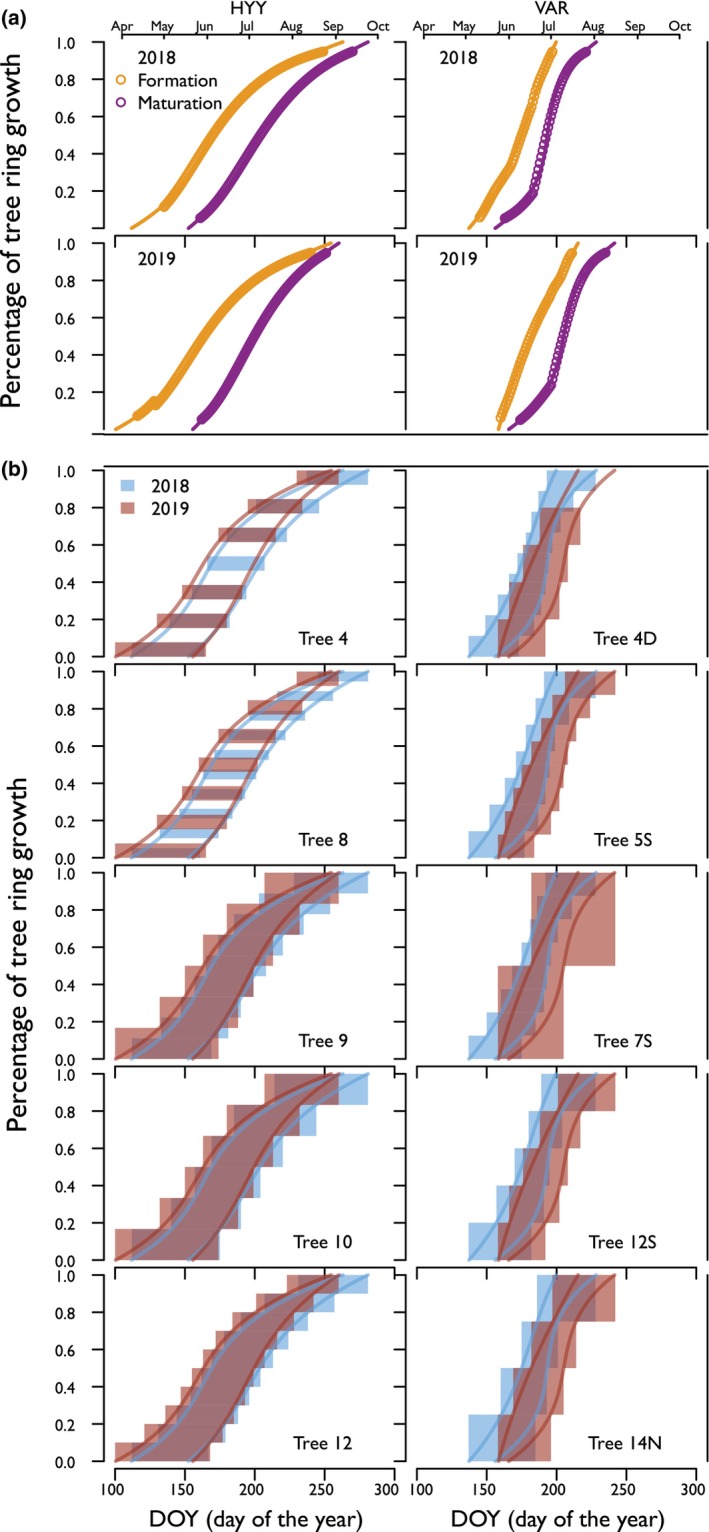
Intra‐annual tree ring growth models for individual trees at Hyytiälä (HYY) and Värriö (VAR) sites for 2018 and 2019. The top panels (a) illustrate the modeled cell formation and maturation phases of radial growth for 2018 (yellow) and 2019 (purple) at the site level. The lower panels (b) show the percentage of tree ring growth (*y*‐axis) over the Day of Year (DOY) (*x*‐axis) for individual trees, with the red and blue boxes representing the temporal window of formation and maturation of the thin sections analyzed from each tree ring (Supporting Information Notes S3).

Thin sections (*c*. 80‐μm‐thick tangential cuts relative to the growth direction) were obtained from the tree rings of five individual trees at each site using a cryo‐microtome (Notes S1). This thickness was chosen to provide sufficient material for isotopic analysis (Belmecheri *et al*., 2022). All thin sections were analyzed for isotopic composition, except for trees 4 and 8 at HYY, which had many subsections (Fig. 2). For these trees, only odd‐numbered subsections were analyzed due to the substantial number of thin sections (≥ 14).

In this study, we analyzed resin‐extracted wood samples to investigate the isotopic composition of the tree rings. The wood samples underwent solvent extraction using a mixture of ethanol and toluene to remove resins, oils, and other extractives that could interfere with the isotopic signals. After extraction, the wood was thoroughly dried before isotopic analysis.

Although cellulose is often used in isotopic analyses due to its chemical stability and simpler chemical connection to the sucrose from the phloem, resin‐extracted wood provides comparable and valuable insights (Rinne‐Garmston *et al*., 2023).

Studies have demonstrated a high covariation between the isotopic compositions of cellulose and resin‐extracted wood, indicating that resin‐extracted wood can reliably reflect the isotopic composition found in cellulose, although there is an offset in the δ^18^O values of these materials (Gori *et al*., 2013; Weigt *et al*., 2015). Nevertheless, even though the signals in wood and cellulose δ^18^O covary, there are differences in how these signals relate to environmental factors, and they may in fact respond to different climatic variables (Sidorova *et al*., 2008). Synthesis of the current knowledge in Helle *et al*. (2022) suggests that cellulose may be a more robust proxy for environmental variables, especially for time series where long sections, containing both hardwood and sapwood, are utilized (see Helle *et al*., 2022 and references therein), whereas wood δ^18^O analysis should be constrained to sapwood while being conscious of the complicated wood chemistry. Our main objective was to understand the isotopic composition of all fixed organic material in the wood during growth, encompassing a broader spectrum of organic compounds beyond cellulose, as the lignin also comes from the current carbohydrates during wood formation (Gori *et al*., 2013; Weigt *et al*., 2015; Leppä *et al*., 2022; Rinne‐Garmston *et al*., 2023). Therefore, our approach focuses on examining the isotopic composition of the wood tissue as a whole (excluding resins and mobile phases), while acknowledging that interpretation of the wood δ^18^O signal may not be as straightforward as that in cellulose.

For δ^18^O analyses, we used a flash IRMS elemental analyzer operated in pyrolysis mode with a glassy carbon reactor at 1400°C, coupled to a Delta V Plus IRMS in the laboratory at the University of Basel. Samples were introduced using a Costech Zero‐Blank Autosampler (NC Technologies Srl, Milan, Italy), which fed them into the pyrolysis reactor. Values were normalized to the Vienna Standard Mean Ocean Water/Standard Light Antarctic Precipitation scale using calibrated in‐house standards with δ^18^O values of +2.89‰, +8.91‰, and +23.96‰. The long‐term analytical precision of our measurements is 0.2‰ (Notes S2).

### Data analysis

#### Determination of the growth periods for each tree ring subsection

Yearly tree‐ring growth curves for our study sites and years have been examined in Tang *et al*. (2022). In brief, the start date of tracheid production and the end date of tracheid maturation for a given ring location were simulated via a dynamic growth model, *Carbon Allocation Sink Source Interaction* (CASSIA), which was calibrated against weekly xylogenesis observations from micro‐core analysis (Tang *et al*., 2022). Based on the growth curves, the period from the start of tracheid production to the end of tracheid maturation was determined as the growth period for a given tree ring subsection (Morino *et al*., 2021; Martínez‐Sancho *et al*., 2022; Perez‐de‐Lis *et al*., 2022) (Fig. 2). We assigned each section's last maturation day to integrate the growth timing and duration into the correlation analysis. We correlated it with the corresponding daily RH data. We then worked our way back in time, integrating the mean values of RH over the entire tracheid formation and maturation period to correlate with the measured δ^18^O values for the corresponding thin section. In this study, we did not account for differences between trees in our correlation analysis, as each tree was analyzed independently, allowing us to capture each tree's unique growth and δ^18^O patterns.

#### Micrometeorological data

We utilized data on forest ecosystem gas exchange and meteorology to examine the relationships between the isotopic data, tree function, environmental variables, and gross primary productivity (GPP, Fig. 1) (Mammarella *et al*., 2016; Kulmala *et al*., 2019). Although sugar sampling was conducted between 13:00 h and 16:00 h for practical reasons, the isotopic composition in the phloem and wood reflects an integration of environmental conditions over a broader period. Therefore, we focused on integrating the micrometeorological data for the daytime hours between 09:00 h and 15:00 h, capturing the period when assimilation and evaporative processes are active and can influence water at the leaf level. To facilitate comparisons with the δ^18^O isotope values collected during 2018 and 2019, a running integration of 1, 5, 10, 15, 20, 25‐days was employed for all available variables from the eddy covariance stations, including rainfall, evapotranspiration (E), GPP, temperature, and RH (Aalto *et al*., 2023, 2014). These integrations were aligned to ensure that the integration period captured the average environmental conditions the trees were experiencing during the measurement day or period when it came to tree ring subsections. Subsequently, the δ^18^O values were paired with the micrometeorological dataset based on the dates, enabling correlation analyses between δ^18^O and critical variables such as RH.

#### Approximations to the proportion of ^18^O exchange during wood formation

Cellulose δ^18^Ο values can be approximated as (Roden *et al*., 2000):

(Eqn 1) $$\delta^{18}O_{\text{cell}}=p_{\mathrm{ex}}\left(\delta^{18}O_{\mathrm{xw}}+\varepsilon_{c}\right)+\left(1-p_{\mathrm{ex}}\right)\left(\delta^{18}O_{\mathrm{lw}}+\varepsilon_{c}\right)$$

where δ^18^O_cell_ was approximated to δ^18^O_wood_ + 4.4‰ (based on data not published from SILL), δ^18^O_xw_ is the measured source water (i.e. xylem or twig water/extracted stem water), $\varepsilon_{c}$ is the oxygen isotope fractionation between water and sugar, and δ^18^O_lw_ is water at the needle or evaporation site. *p*_ex_ describes the proportion of ^18^O exchange with the surrounding water during cellulose synthesis in the stem (Barbour & Farquhar, 2000). By rearranging Eqn 1, we can solve for *p*_ex_ using the canonical value of 27‰ for $\varepsilon_{c}$ (Sternberg & DeNiro, 1983; Sternberg & Ellsworth, 2011) that refers to equilibrium fractionation for carbonyl oxygen at the site of cellulose synthesis in the stem. As our approach uses whole wood, including both cellulose and lignin, we also estimated an apparent pex (*p*_ex.app_) derived from wood, sugar, and source and leaf water observations.

(Eqn 2) $$p_{\mathrm{ex}}=\frac{\delta^{18}O_{\text{cell}}-\delta^{18}O_{\mathrm{lw}}-\varepsilon_{c}}{\delta^{18}O_{\mathrm{xw}}-\delta^{18}O_{\mathrm{lw}}}$$

(Eqn 3) $$p_{\mathrm{ex}.\mathrm{app}}=\frac{\delta^{18}O_{\text{sugar}}-\delta^{18}O_{\text{wood}}}{\delta^{18}O_{\mathrm{lw}}-\delta^{18}O_{\mathrm{xw}}}$$

This approximation might contain some temporal mismatch between the co‐occurring sugar and leaf water residence time in the leaf and between the leaf and stem materials. Thus, we address potential time lags to mitigate interpretive uncertainty by considering the importance of aligning the measured δ^18^O values for both resin‐extracted wood and leaf water to specific weekly integrations. By standardizing these values at specific time points, our analysis seeks to provide a perspective on understanding the differences between *p*_ex_ and *p*_ex.app_ and its effect and implications for interpreting wood's isotopic composition, thereby contributing to a better understanding of δ^18^O signal integration in tree rings and its climatic interpretations.

### Statistical analyses

**Fig. 3:**
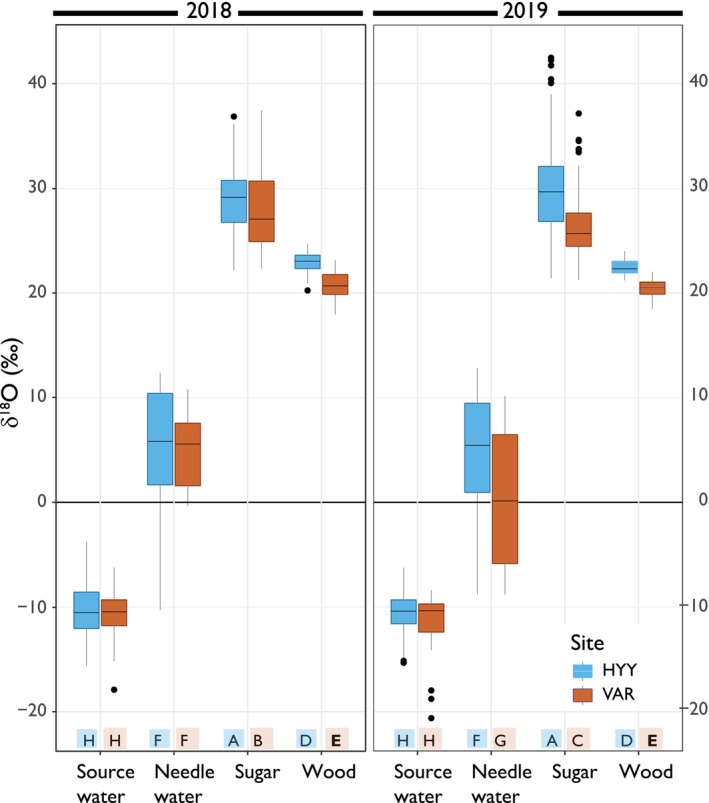
Isotopic distributions of various δ^18^O values from water and carbohydrate pools for 2018 and 2019 studied in Hyytiälä (HYY) and Värriö (VAR). The *x*‐axis denotes the main pools, while the *y*‐axis represents the complete δ^18^O scale and whiskers extending to 1.5 times the interquartile range. The different colors indicate different sites. Based on Tukey's honest significant difference test, groups with the same letters are not significantly different in their δ^18^O values – similar visualization in boxplot based on each variable in Supporting Information Fig. S8.

The dataset consisted of multiple groups of δ^18^O measurements, each representing a distinct isotopic pool. These measurements included δ^18^O of source water (rainwater, soil water, and twig water); leaf water enrichment reflecting the isotopic composition in N0 and N1 needles; δ^18^O of WSCs in phloem, needles, and roots; and δ^18^O in wood. When statistical analysis showed no significant differences among the sub‐pools within each category, we combined them for further analysis. We conducted a one‐way ANOVA to test whether the group means were statistically different, followed by Tukey's honest significant difference (HSD) test for pairwise comparisons between the groups (Fig. 3). The HSD test results assigned letters to each group to indicate statistical similarity. To address potential issues of unequal variances and sample sizes, we also applied the Games‐Howell test and supported the robustness of our results (Figs S8–S11). Because this study focuses on tracing the isotopic composition from the leaf to the wood, we implemented a correction for the WSCs to mitigate isotopic effects caused by sugar alcohols such as pinitol, which is a WSC not used in wood synthesis. This correction allowed us to more accurately represent the isotopic composition of the compounds that contribute to wood formation. For this correction, we assumed a stable δ^18^O value of 25‰ (Leppä *et al*., 2022).

For a significant part of our dataset, we obtained pinitol concentration data and found no correlation between pinitol concentration and WSC δ^18^O. Using the pinitol concentration, we performed a mass balance correction to adjust the WSC values by accounting for pinitol influence on the WSCs. In cases where measured pinitol concentration was unavailable, we used the average concentration observed in our dataset (Notes S4). From now on, the pinitol‐corrected δ^18^O values derived from WSCs will be referred to as ‘sugars’ in this paper (Fig. S12). We conducted a correlation analysis for the complete dataset to examine the relationships between the different δ^18^O pools and their corresponding time. This approach leveraged the close relationship between the data distributions and increased the degrees of freedom in the face of limited observations after the time aggregation windows. The data were aggregated at weekly and monthly resolutions to synchronize them with the environmental variables on the same temporal resolution. Subsequently, correlation matrices were computed and statistically evaluated to validate the significance of the relationships. The significance level was set at 0.05, differentiating any relationships that failed to meet this criterion.

We conducted a correlation analysis to test the hypothesis that RH affects δ^18^O pools in tree components with different temporal integrations. This analysis, guided by the observations in Leppä *et al*. (2022) and Tang *et al*. (2023), ranged from short (1–5 d) to long (up to 25 d) integration periods, focusing on how these periods correlate with δ^18^O values in needle water, sugars, and wood (Notes S5).

## Results

### General isotopic composition of water, sugar, and wood pools

During the 2018 and 2019 growing seasons, distinct patterns of δ^18^O variability were observed, from source water, through needle water and sugars, to wood at both HYY and VAR sites (Fig. 3). This variability was influenced markedly by the different processes and periods these pools integrated during the growing seasons. Additionally, the trees responded to environmental fluctuations throughout the seasons (Fig. 1), leading to pronounced seasonal and daily variations in δ^18^O values, especially evident in the evaporative enrichment of needle water (Table 1).

**Table 1:** Seasonal variance, SD, and seasonal mean δ^18^O values for different pools (needle water, source water, sugar, and wood) at Hyytiälä (HYY) and Värriö (VAR) sites.

δ^18^O pool	Site	Variance	SD (‰)	Mean (‰)	HSD test
Source water	HYY	4.39	2.1	−10.55	b
Source water	VAR	4.76	2.18	−10.89	b
Needle water	HYY	37.17	6.1	3.81	a
Needle water	VAR	32.11	5.67	2.59	a
Sugars	HYY	11.22	3.35	29.32	c
Sugars	VAR	10.66	3.27	27.09	d
Wood	HYY	0.82	0.91	22.68	e
Wood	VAR	1.65	1.28	20.58	f

The homogeneous subsets (HSD) test results are also included, indicating statistical distinctions between the groups combining both seasons. The data provide a quantitative analysis of δ^18^O variation within each pool across the sites, reflecting the site‐specific environmental and physiological influences on isotopic composition.

In both years and at both sites, we observed consistent patterns in δ^18^O values for source water and needle water, with the latter influenced by evaporative enrichment (Fig. 3). At the source level – soil water, precipitation, and twig water – the δ^18^O values showed a range of variation (i.e. difference between maximum and minimum values) of 11.7‰ and 13.2‰ in HYY and VAR, respectively. After evaporative enrichment at the needle level, this range increased to 29.3‰ at HYY and 19.6‰ at VAR. However, the δ^18^O range decreased in sugars, dropping to 14.9‰ in HYY and 12.8‰ in VAR. In wood, the range decreased further to 4.5‰ and 5.8‰, respectively.

### Source water pool

**Fig. 4:**
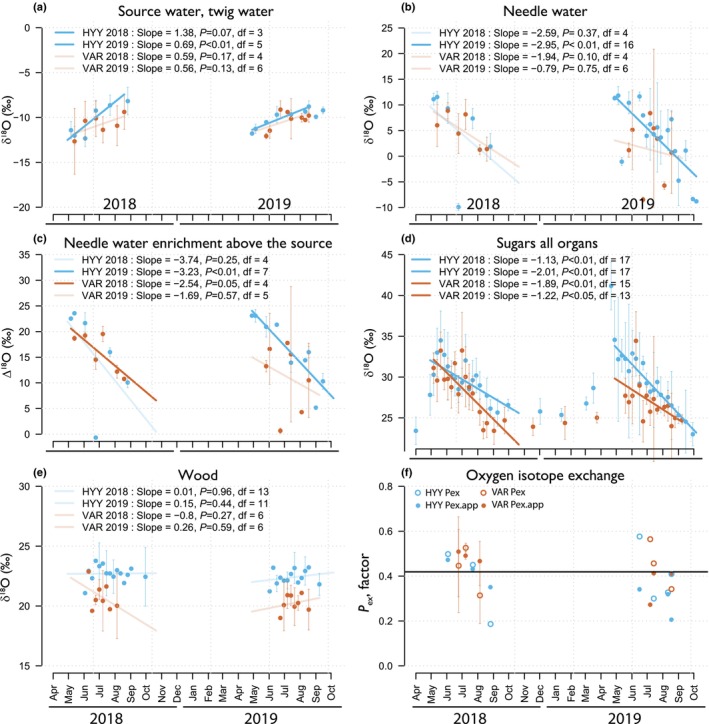
Seasonal trends in a weekly aggregation of oxygen isotopes across various pools and sites. The six subplots share an *x*‐axis that marks the temporal window of data collection as the initials of the months for each year (error vertical lines show a 95% CI). The *y*‐axis represents the full δ^18^O scale, and the *y*‐axis of ‘f’ shows the exchange factor (*p*_ex_) using Eqns 2 and 3. Blue and orange colors signify data from Hyytiälä (HYY) and Värriö (VAR) sites, respectively. Plot (a) focuses on δ^18^O in source water (measured in twigs), while plot (b) shows needle water after evaporative enrichment. Plot (c) presents isotopic needle water enrichment above source water; plot (d) covers δ^18^O in leaf and phloem sugars; plot (e) δ^18^O in tree ring sections; and plot (f) shows the *p*_ex.app_ factor. transparent trend lines are trends with *P* values larger than 0.05, so the trend is not significant. Note that in (f) we do not report the regression and slopes due to the reduced number of estimates, or df is too low.

Our analysis of the source water pools – comprising soil water, precipitation, and twig water – revealed a notably consistent isotopic signal across these pools throughout the seasons. As illustrated in Fig. 3, the source water δ^18^O variability is similar among sites and years (Fig. S8). The general uniformity among sites and years showed that twig water has an overall increasing trend in the δ^18^O of source water from June to September during the growing seasons (Fig. 4).

### Needle water pool

The variability in needle water δ^18^O values shows the evaporative effects. We found no significant difference between the δ^18^O distributions of needle water between sites or years (Table 1; Fig. 3). However, an exception was observed in July 2018 at HYY, where the needle water had very low values during a period of rainfall (Fig. 4b,c). The overall pattern across the season shows a decrease in needle water δ^18^O values from May to August (Fig. 4).

### Sugar pool

**Fig. 5:**
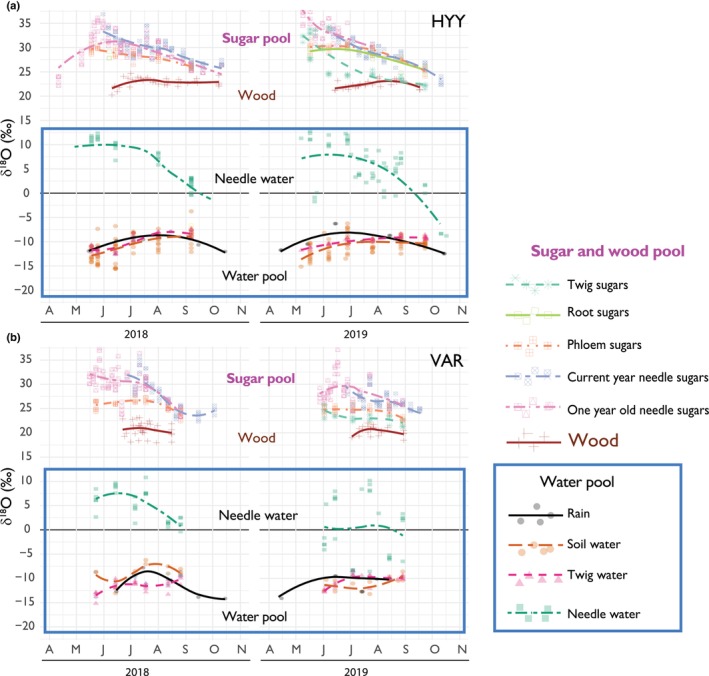
Intra‐annual δ^18^O variations in source water, needle water, sugars, and wood at (a) Hyytiälä (HYY) and (b) Värriö (VAR) sites during 2018 and 2019, plotted in δ^18^O space. Fitted smoothed splines illustrate the seasonal profiles for each pool.

The δ^18^O values of all sugar pools followed a clear seasonal trend, mirroring the mean pattern of needle water enrichment, as shown in Fig. 4. Both sites and years exhibited a declining trend throughout the growing season (Fig. 4d). In 2019, twig sugars exhibited overall values that were 4‰–5‰ lower than those of the other sugar pools at both sites (Fig. 5). Additionally, no significant differences were found in the δ^18^O values of sugar pools at HYY between the 2 yr (Fig. 3). Conversely, VAR sugar pools showed significant annual differences in δ^18^O values, likely reflecting distinct environmental influences at this location (Fig. 3). This was the annual pattern; however, the seasonal variation was sensitive to environmental changes, as shown in Fig. 4.

### Tree ring pool

The intra‐annual wood sections from 2018 and 2019 exhibited significantly lower variance, nearly an order of magnitude less, compared to the sugar sources during the growing season (Table 1). Furthermore, the seasonal trends in the wood were less pronounced than those observed in the sugar pools (Fig. 4e), indicating a marked reduction in the seasonality in wood samples at both sites (Fig. 5). However, Figs 3 and 4(e) show differences in the wood δ^18^O mean values between our two sites. VAR shows lower values than HYY. Moreover, Fig. 4(f) shows that average *p*_ex.app_ in VAR is slightly higher than HYY (0.42 in VAR vs 0.36 in HYY), reflecting the observed differences in wood δ^18^O (Fig. 4e). The comparison of the seasonal trends for sugar and wood pools, particularly from June to September across sites and years, reveals significant differences (*F*‐statistic is 33.56, and the *P*‐value is 0.0012), indicating a disparity in the seasonal slopes between sugar δ^18^O and wood δ^18^O, with sugar δ^18^O showing a steeper slope. For example, in HYY in 2018, the decreasing slope for sugars (−1.13‰ × month^−1^, *P* = 0.001) is significantly steeper than that of wood (0.0085‰ × month^−1^, *P* = 0.959), indicating a pronounced decrease in sugar δ^18^O compared to the relatively flatter slope of wood δ^18^O during this period. This trend continues in HYY for 2019, with sugar exhibiting a more negative slope (−2.01‰ × month^−1^, *P* < 0.0001) than wood (0.15‰ × month^−1^, *P* = 0.44), emphasizing a consistent pattern of more significant variability in sugar δ^18^O, as shown in Table 1. Similarly, in VAR during 2018, the sugar δ^18^O slope (−1.89‰ × month^−1^, *P* = 0.0001) is larger in its absolute value than that of wood δ^18^O (−0.079‰ × month^−1^, *P* = 0.276). This pattern is mirrored in 2019, where the slope for sugar (−1.22‰ × month^−1^, *P* = 0.0303) remains larger than for wood (−0.262‰ × month^−1^, *P* = 0.59). These observations indicate a general pattern that sugar δ^18^O is more sensitive to the seasonal trends in RH, while wood δ^18^O remains relatively more stable (flat) across the growing season. We applied linear models to explore the observed opposing trends between source water and sugars and assess whether they were statistically significant. This approach served only as a tool to detect the presence of these trends and was not intended to imply that the processes themselves follow strict linearity or provide predictive power for future conditions.

### Weekly and monthly correlation analysis of δ^18^O between all pools

**Fig. 6:**
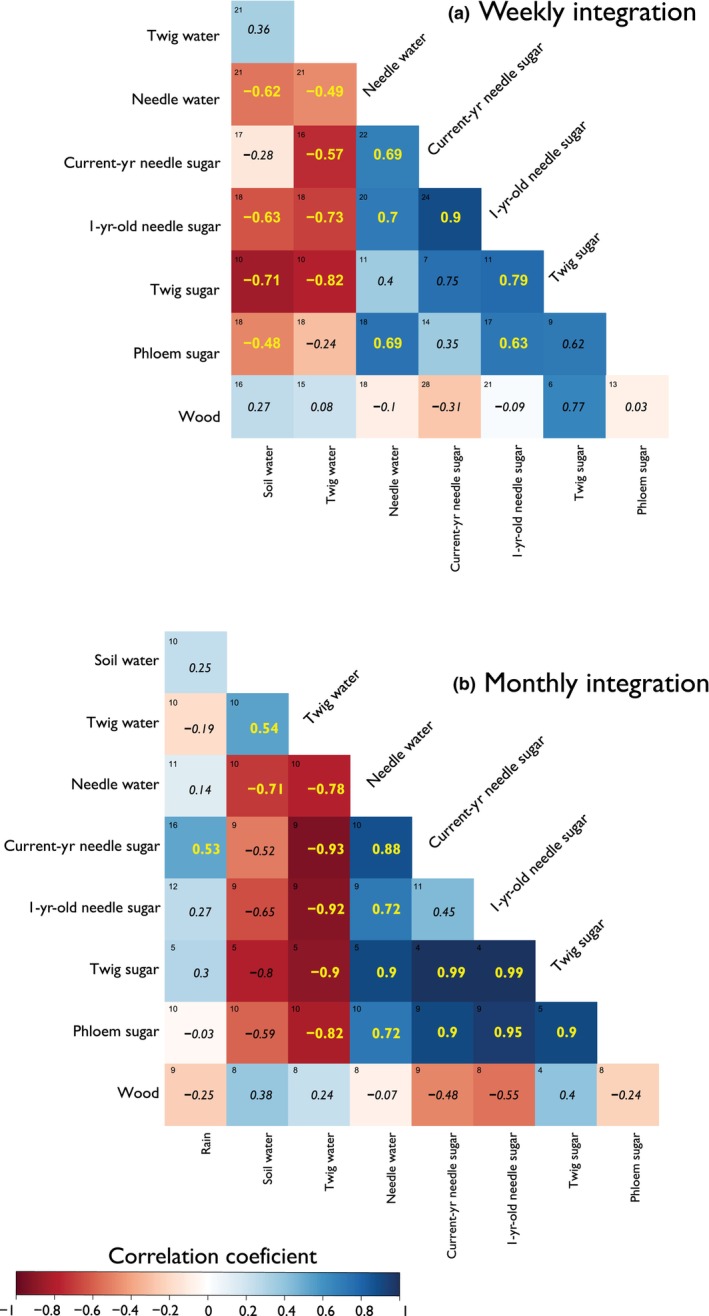
Correlation matrices for oxygen isotope values in different pools. The top matrix (a) represents weekly integration data combining sites and years, while the bottom (b) represents monthly integration data. The color of the boxes indicates the Spearman correlation coefficient, with values in black denoting nonsignificant correlations (*P* > 0.05), and in the upper corner of each correlation square is shown the number of values used in the correlation analysis, as they match the week or month of observation.

The dataset integrated on a weekly resolution revealed a negative correlation between soil water and needle water (*r* = −0.62, *P* = 0.01), N1 sugars (*r* = −0.63, *P* = 0.005), twig sugars (*r* = −0.71, *P* = 0.002), and phloem sugars (*r* = −0.48, *P* = 0.04) (Fig. 6a). The high correlation between twig water and soil water is expected due to their spatial and temporal proximity. In addition, a negative relationship was observed between source water and sugar pools. For example, twig water had significant negative correlations with N0 sugars (*r* = −0.57, *P* = 0.0009), N1 sugars (*r* = −0.73, *P* = 0.001), as well as twig sugars (*r* = −0.82, *P* = 0.007). Evaporative enrichment of the needle water had strong positive correlations with N0 sugars (*r* = 0.69, *P* = 0.0004), N1 sugars (*r* = 0.70, *P* = 0.0005), and phloem sugars (*r* = 0.69, *P* = 0.0003). There was also a strong correlation between the δ^18^O of the N0 sugar and N1 sugar pools (*r* = 0.9, *P* < 0.0001).

The data from twig sugars, especially for HYY in 2019, displayed a slight offset compared to other sugar measurements (Fig. 5). However, it showed a positive correlation with N1 sugar (*r* = 0.79, *P* = 0.004) and a negative overall correlation between sugars and source water. This negative relation is expected from the opposing trends from the water pools and the effects of δ^18^O enrichment. Additionally, the weekly integration correlation analysis shows no significant correlations between the intra‐annual subsections of wood and the other pools, with the closest correlation observed between twig sugars and wood (*r* = 0.77, *P* = 0.16).

Moreover, when examining correlations using a monthly integration approach (Fig. 6b), notable shifts are observed in the correlation coefficients compared to the weekly resolution analysis (Fig. 6a). For instance, the negative correlations between source water and sugar δ^18^O for N1 (*r* = −0.92, *P* = 0.02), twig sugar (*r* = −0.9, *P* = 0.02), and phloem sugar (*r* = −0.82, *P* = 0.03) exhibit higher coefficients on a monthly scale (Fig. 6b). Additionally, twig sugar positively correlates with N0 sugar (*r* = 0.99, *P* = 0.001). Needle water, isotopically enriched, correlates positively (*r* = 0.88, *P* = 0.001) with N0 sugar. In contrast to the weekly integration, a negative correlation (*r* = −0.55, *P* = 0.06) is found between δ^18^O in wood and N1 sugar in the monthly dataset. Although these differences may reflect temporal integration dynamics, the lower sample size for monthly integration reduces its precision, warranting caution when directly comparing absolute coefficients.

### Relationship between RH and δ^18^O pools

**Fig. 7:**
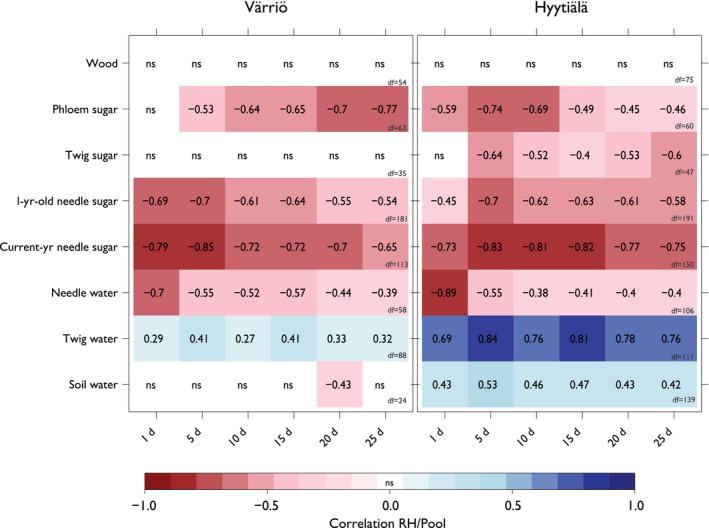
Correlation matrices between relative humidity (RH) for different integration periods and δ^18^O values measured in different pools (source water, needle water, sugars, and wood) for Värriö (VAR) and Hyytiälä (HYY). The color gradient represents the Pearson correlation coefficient *P* < 0.05 and ‘ns’ values with *P* ≥ 0.05. The *x*‐axis displays the various RH integration periods (1, 5, 10, 15, 20, and 25 d), while the *y*‐axis lists the different water and sugar δ^18^O pools. The df values are the degrees of freedom related to the correlation value for each variable.

The correlation analysis exploring the relationship between RH and the δ^18^O values across the water and carbohydrate pools in Fig. 7. RH was calculated using average daytime values integrated over 1, 5, 10, 15, 20, and 25 d, including and leading up to the day of sample collection.

The data in Fig. 7 shows correlation values and the number of integration days of RH across the different pools. For VAR, needle water shows the strongest negative correlation for the shortest integration period, 1 d (*r* = −0.7, *P* < 0.05). Conversely, the strongest negative correlations for needle sugars are observed with a longer integration period of 5 d. However, for phloem sugars, the correlation coefficient increases from the 5‐d integration (*r* = −0.53, *P* < 0.05) to the 25‐d period (*r* = −0.77, *P* < 0.05). At HYY, needle water displays a pattern like VAR, showing the strongest negative correlation at a 1‐d integration period (*r* = −0.89, *P* < 0.05), which then weakens over longer periods. For all sugar pools at HYY, paralleling the trend seen in VAR for needle sugars, the most significant correlations are also observed at the 1–5‐d integration period (scatterplots of these relationships in Figs S13, S14).

## Discussion

During the growing seasons of 2018 and 2019, we investigated the seasonal δ^18^O dynamics of source water, needle water, sugars, and wood at HYY and VAR to understand how these different pools influence the δ^18^O values of tree rings. Our measurements revealed distinct patterns of δ^18^O variability across these pools, markedly influenced by environmental fluctuations and physiological processes (Fig. 3). Notably, we observed opposing seasonal trends in δ^18^O values for sugars and source water, with source water δ^18^O increasing while sugar δ^18^O decreased, in line with leaf water patterns. Moreover, opposing trends lead to a flattened intra‐annual δ^18^O profile in the tree rings. This flattening effect arises from two key factors: the isotope exchange between sugars and source water during wood formation and the impact of their contrasting seasonal δ^18^O patterns. Thus, understanding these intra‐annual δ^18^O variations is crucial because different seasonal variables significantly shape the isotopic signals recorded in tree rings (Belmecheri *et al*., 2022). Studies have demonstrated that our approach using resin‐free wood reliably captures the climate variation recorded in δ^18^O. Strong correlations between resin‐free or whole wood and cellulose δ^18^O measurements have been observed, with a consistent positive offset which was taken into consideration in our analysis (Barbour *et al*., 2001; Szymczak *et al*., 2011; Roden & Farquhar, 2012; Gori *et al*., 2013; Mischel *et al*., 2015; Weigt *et al*., 2015; Riechelmann *et al*., 2016; Guerrieri *et al*., 2017). Our findings highlight the importance of focusing on the seasonal processes that influence isotopic signatures, providing valuable insights for refining predictive models and enhancing our understanding of tree physiology and climate interactions on intra‐annual scales.

### Increasing seasonal trend of source water δ^18^O

Throughout the growing seasons, our observations of source water δ^18^O – including soil water and twig water – closely mirrored the δ^18^O of precipitation, indicating that trees at both HYY and VAR are predominantly using recent precipitation as their water source (Fig. 5). The seasonal increase in source water δ^18^O from early spring to late summer corresponds with the δ^18^O of precipitation during these periods (Kortelainen & Karhu, 2004; Saurer *et al*., 2016; Nelson *et al*., 2021) (Figs 3, 5). This consistency suggests that, at our sites, the isotopic composition of source water used by trees during the growing season is primarily determined by contemporary precipitation recharging the active soil layers. Despite the challenges in soil water sampling at VAR due to the rocky terrain, the observed patterns in twig water δ^18^O reinforce this conclusion.

During the growing season both source water δ^18^O and RH increase throughout most of the growing season (Figs 1, 4a). The positive correlation observed in Fig. 7 can be explained by rainfall during the growing season increasing RH and by rising temperatures from spring to summer leading to higher δ^18^O values in rainfall. Although RH below saturation generally promotes soil surface water evaporation – resulting in enriched water δ^18^O – the relationship between the water that trees uptake and environmental drivers is more complex and appears to be more closely related to the δ^18^O of precipitation. However, the observed positive relationship between RH and source water δ^18^O at our study site likely arises from the combined effects of rainfall and temperature (Fig. S15), which together drive coherent seasonal trends between source water, RH, and temperature (Kortelainen, 2007).

### Decreasing seasonal trend in sugars and needle water δ^18^O

Our research indicates that needle water δ^18^O is sensitive to short‐term fluctuations in RH, resulting in a robust daily correlation (Fig. 7) (Cernusak *et al*., 2005; Barnard *et al*., 2007; Gessler *et al*., 2009; Fiorella *et al*., 2022; Leppä *et al*., 2022). This response underscores the potential of needle water isotopic composition as an indicator for tracking rapid atmospheric changes (Dongmann *et al*., 1974). The strongest correlations between needle water δ^18^O and RH were observed over a 1‐d integration period, suggesting that sampling of needle water should capture daily variations and be conducted as frequently as feasible to accurately represent environmental processes in Scots pine at these sites.

The influence of RH was also evident in needle sugars, which integrated changes over slightly longer periods, showing stronger correlations with RH over 1 to > 5 d due to the larger needle sugar pool (Leppä *et al*., 2022). This integration suggests that while needle sugar sampling can be less frequent than needle water, it should still consider the temporal dynamics of RH to accurately reflect environmental drivers (Leppä *et al*., 2022).

**Fig. 8:**
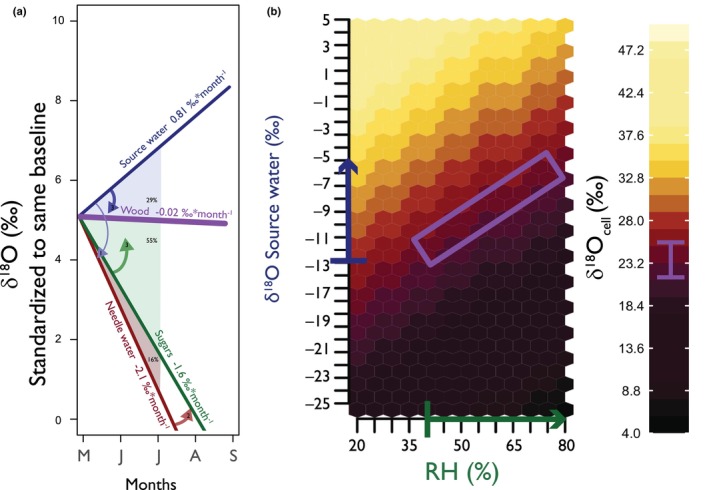
Synthesis of the seasonal δ^18^O patterns from source water through needle water to sugars and wood. (a) This conceptual figure illustrates the opposing seasonal trends of δ^18^O integrated through various tree water and carbon pools, starting from source water and progressing through needle water, sugars from twigs and phloem, and finally wood. The slopes represent the mean rates of change (‰ month^−1^) in δ^18^O observed in these pools, derived from Fig 4(a,b,d,e). The *y*‐axes depict an arbitrary δ^18^O baseline adjusted for each component for this graphical purpose only. However, the average baseline values of −10‰ for source water, 5‰ for needle water, 30‰ for sugars, and 20‰ for wood. The area of each triangle shows the proportion of each triangle with respect to the sum of the three areas. The arrows show the mechanistic direction and order from source to needle, water to sugar to wood. These site‐specific results may differ in regions with different climate regimes or seasonal patterns, highlighting the importance of local conditions when interpreting δ^18^O signals in tree tissues. (b) Heat map illustrating how the same concurrent trends in source water δ^18^O and relative humidity (RH) can offset one another, producing a consistent result in δ^18^O cellulose using Eqn 1. As source water becomes more enriched and RH increases, the two effects balance out, resulting in relatively stable δ^18^O signals in the final tissue.

As sugars move from needles to the phloem, **s**everal factors might contribute to the observed marginally lower δ^18^O values in phloem sugars compared to needle sugars (Fig. 5). One possible explanation is isotopic exchange during phloem loading, where sugars may exchange oxygen atoms with source water, leading to a reduction in δ^18^O values in the phloem sugars (Gessler *et al*., 2014). Additionally, observations by Fiorella *et al*. (2022) indicate that most sugars loaded into the phloem come from the base of the needle, an area less affected by evaporative enrichment than the needle tip. This spatial variation means that sugars entering the phloem are inherently lower in δ^18^O compared to those synthesized at the needle tip (Barbour & Farquhar, 2000; Helliker & Ehleringer, 2002; Kannenberg *et al*., 2021; Fiorella *et al*., 2022). Furthermore, there is a difference in the temporal integration of the samples: phloem sugars integrate over *c*. 5 d (Tang *et al*., 2023), capturing a broader range of environmental conditions, while twig sugars were collected during the afternoon, a period characterized by higher evaporative enrichment due to drier atmospheric conditions. This timing likely results in higher δ^18^O values in twig sugars compared to the phloem sugars, which represent a long‐term average. Consequently, the seasonal δ^18^O trend in phloem sugars is less negative than in needle water (Fig. 8), with the most significant variations appearing early in the growing season, especially at the VAR site. As the season progresses, the δ^18^O values of needle and phloem sugars converge (Fig. 5), probably indicating the increasing RH effect on the overall signals that integrate in different pools.

### Dampened seasonal trend on intra‐annual δ^18^O tree rings

Our observations demonstrate that the opposing seasonal trends between evaporative δ^18^O enrichment fixed in sugars and the source water δ^18^O are key factors shaping the isotopic composition of intra‐annual tree rings (Roden *et al*., 2000; Monson *et al*., 2018; Belmecheri *et al*., 2022; Leavitt & Szejner, 2022; Vitali *et al*., 2023). The δ^18^O values we observe in the intra‐annual tree‐ring subsections represent the integration of both source water and phloem sugars from the time of cell formation to the completion of cell maturation (Fig. 2) (Rinne *et al*., 2015; Belmecheri *et al*., 2018; Tang *et al*., 2022). When source water δ^18^O and RH are positively correlated (Figs 7, 8), their effects can offset each other and reduce the overall variability in δ^18^O in wood, as seen in our data (Figs 4, 5). Conversely, a negative correlation (e.g. enriched source water with lower RH) could amplify the δ^18^O recorded in the wood, resulting in higher variance recorded in the wood (Fig. 8b). Additionally, if one of these two factors remains relatively stable, such as a homogeneous source water or nearly constant RH, the influence of the other factor would become more evident in tree ring records (Fig. 8). This seasonal dynamic could become even more apparent under fluctuating aridity, where changes in RH drive greater evaporative enrichment at the leaf level and, in turn, increase the sensitivity of δ^18^O in needle water, sugars, and intra‐annual tree ring subsections (Kahmen *et al*., 2011). The relative influence of evaporative enrichment vs source water thus emerges as a fundamental factor in understanding the variance captured in tree‐ring δ^18^O records. Therefore, theoretically, by analyzing tree ring subsections, we can constrain these sources of variability and gain insights into past climate patterns and long‐term changes at a seasonal resolution (Gessler *et al*., 2014). However, this dynamic interplay between ^18^O‐enriched sugars and source water may vary across environments, highlighting the importance of considering local seasonality that can influence the intra‐annual isotopic composition in tree rings (Xu *et al*., 2020; Szejner *et al*., 2021; Giraldo *et al*., 2022).

Additionally, the integration of δ^18^O signals in woody biomass is influenced by the exchange of oxygen atoms between sugars and source water during wood formation *p*_ex_ (Song *et al*., 2014). Although *p*_ex_ is often assumed to be *c*. 42% (Roden *et al*., 2000), several studies suggest that the assumption of a constant *p*_ex_ may vary with environmental conditions affecting physiological processes (Cheesman & Cernusak, 2017; Szejner *et al*., 2020; Martínez‐Sancho *et al*., 2023; Bailey *et al*., 2025). Morgner *et al*. (2024) further proposed that increases in atmospheric CO₂ could alter source – sink dynamics, potentially shortening turnover times and thereby influencing *p*_ex_. The observed differences between sites suggest a potential relationship between oxygen exchange during wood formation and environmental conditions (Cheesman & Cernusak, 2017; Szejner *et al*., 2020; Martínez‐Sancho *et al*., 2023). The slightly higher *p*_ex.app_ values at VAR indicate a more active exchange between source water and sugars during wood formation, possibly due to site‐specific environmental factors such as lower temperatures or longer wood maturation periods and slower carbon turnover (Song *et al*., 2014). This pattern aligns with conditions typically found in more extreme sites like VAR. Although our focus in this investigation is on intra‐annual variation, future research on post‐photosynthetic fractionation processes (e.g. *p*_ex_, and the use of old reserves (Gessler & Ferrio, 2022)) will be important for improving the long‐term δ^18^O‐based climate reconstructions (Holloway‐Phillips *et al*., 2023; Wieloch *et al*., 2024). Therefore, a deeper understanding of these processes can help refine both the sampling strategies and the interpretation of δ^18^O signals in tree rings under diverse environmental conditions.

### Conclusions

Our study aimed to clarify the intricate relationship between sugar and source water δ^18^O values and their impact on the seasonal isotopic composition of tree rings. We observed that opposing seasonal trends between these components tend to neutralize each other, resulting in a relatively uniform seasonal isotopic profile within the tree rings. By tracing δ^18^O variations from source water through needle water and sugars to wood, we illustrated how these signals are integrated and offset during transport and incorporation into woody tissue. This seasonal progression underscores the complex mechanisms through which trees record environmental changes, providing a nuanced understanding of tree physiological responses over the growing season.

Our correlation analyses (Fig. 7) revealed that needle water δ^18^O values are strongly correlated with short‐term fluctuations in RH, particularly over a 1‐d integration period. By contrast, sugars showed stronger correlations with RH over 1–5 d of integration periods. This observation aligns with findings from other studies (Leppä *et al*., 2022; Tang *et al*., 2022) that have reported part of this dataset for isotopic responses in Scots pine using carbon and oxygen isotopes.

When shifting from weekly to monthly integration, the seasonal trends among different oxygen isotope pools exhibited coherent and expected patterns (Fig. 6). This highlights the importance of considering appropriate timescales when interpreting relationships between different components and subsections of tree rings. Our study also emphasized the necessity of accounting for site‐specific environmental conditions when interpreting δ^18^O in wood, highlighting the intricate nature of these processes and the opportunities for further research. The differences in responses between the HYY and VAR sites and the challenges associated with estimating *p*_ex_ or *p*_ex.app_ underscore the challenges of these processes.

By analyzing intra‐annual sections of tree rings, we hope to provide insights into climate patterns and long‐term environmental changes with seasonal resolution. Our findings suggest the importance of examining different sections of the ring, such as earlywood and latewood, in relation to specific research questions. For instance, ring sections formed during periods of variable and low RH may capture greater variability related to water demand, whereas those formed during periods of low variability and high RH might better reflect variability in the source water signal. These insights can be helpful for more precise climate reconstructions and modeling efforts.

## Competing interests

None declared.

## Author contributions

PS and KTR‐G designed the study and data collection and wrote the manuscript. PS and CA performed the data analysis. YT and PS‐A carried out the sample collection. YT also performed data collection and micro‐core analysis. GY conducted the tree‐ring micro‐sectioning. ES, DBN, AK and MS performed the isotope analysis. KTR‐G supervised the project. All authors provided comments on the manuscript.

## Disclaimer

The New Phytologist Foundation remains neutral with regard to jurisdictional claims in maps and in any institutional affiliations.

## Supporting information


**Fig. S1** Timing of water, wood, and sugar sample collection across individual trees at HYY.
**Fig. S2** Continuation. Timing of water, wood, and sugar sample collection across individual trees at HYY.
**Fig. S3** Continuation. Timing of water, wood, and sugar sample collection across individual trees at HYY.
**Fig. S4** Timing of water, wood, and sugar sample collection across individual trees at VAR.
**Fig. S5** Continuation. Timing of water, wood, and sugar sample collection across individual trees at VAR.
**Fig. S6** Continuation. Timing of water, wood, and sugar sample collection across individual trees at VAR.
**Fig. S7** Continuation. Timing of water, wood, and sugar sample collection across individual trees at VAR.
**Fig. S8** Distributions of δ^18^O values for water, sugar, and wood pools in HYY and VAR for 2018 and 2019.
**Fig. S9** Forest plot of pairwise group mean differences with 95% confidence intervals from the Games‐Howell test.
**Fig. S10** Continuation. Forest plot of pairwise group mean differences with 95% confidence intervals from the Games‐Howell test.
**Fig. S11** Forest plot of non‐significant pairwise comparisons with 95% confidence intervals from the Games‐Howell test.
**Fig. S12** Comparison of δ^18^O in water‐soluble carbohydrates before and after correction for pinitol influence.
**Fig. S13** Scatterplots of δ^18^O values from multiple pools vs relative humidity across 1–50 d integration windows at HYY, with a summary heatmap of correlation coefficients.
**Fig. S14** Scatterplots of δ^18^O values from multiple pools vs relative humidity across 1–50 d integration windows at VAR, with a summary heatmap of correlation coefficients.
**Fig. S15** Temporal variability of precipitation, snow depth, and air temperature at HYY and VAR.
**Notes S1** Thin sectioning of the tree rings.
**Notes S2** Isotope analysis for tree ring sections.
**Notes S3** Determination of the periods for each tree ring subsection.
**Notes S4** Pinitol correction on the WSCs.
**Notes S5** Correlations between pools and relative humidity and temporal integration periods.
**Table S1** Total number of samples collected per tree and soil depth at HYY.
**Table S2** Total number of samples collected per tree and soil depth at VAR.
**Table S3** Summary of collection dates, number of trees, and δ^18^O measurements for water, wood, and sugar pools at HYY.
**Table S4** Summary of collection dates, number of trees, and δ^18^O measurements for water, wood, and sugar pools at VAR.
Please note: Wiley is not responsible for the content or functionality of any Supporting Information supplied by the authors. Any queries (other than missing material) should be directed to the *New Phytologist* Central Office.

## Data Availability

Data availability available on zenodo. doi: 10.5281/zenodo.15303368.
