# Supplementary material for: Opposing seasonal trends in source water and sugar dampen intra‐annual variability in tree rings oxygen isotopes
Source: New Phytol. 2025 May 20;247(1):97–114. doi: 10.1111/nph.70223 (PMC12138169; doi:10.1111/nph.70223)
Supplement: Supplementary file 1 — Fig. S1 Timing of water, wood, and sugar sample collection across individual trees at HYY. Fig. S2 Continuation. Timing of water, wood, and sugar sample collection across individual trees at HYY. Fig. S3 Continuation. Timing of water, wood, and sugar sample collection across individual trees at HYY. Fig. S4 Timing of water, wood, and sugar sample collection across individual trees at VAR. Fig. S5 Continuation. Timing of water, wood, and sugar sample collection across individual trees at VAR. Fig. S6 Continuation. Timing of water, wood, and sugar sample collection across individual trees at VAR. Fig. S7 Continuation. Timing of water, wood, and sugar sample collection across individual trees at VAR. Fig. S8 Distributions of δ18O values for water, sugar, and wood pools in HYY and VAR for 2018 and 2019. Fig. S9 Forest plot of pairwise group mean differences with 95% confidence intervals from the Games‐Howell test. Fig. S10 Continuation. Forest plot of pairwise group mean differences with 95% confidence intervals from the Games‐Howell test. Fig. S11 Forest plot of non‐significant pairwise comparisons with 95% confidence intervals from the Games‐Howell test. Fig. S12 Comparison of δ18O in water‐soluble carbohydrates before and after correction for pinitol influence. Fig. S13 Scatterplots of δ18O values from multiple pools vs relative humidity across 1–50 d integration windows at HYY, with a summary heatmap of correlation coefficients. Fig. S14 Scatterplots of δ18O values from multiple pools vs relative humidity across 1–50 d integration windows at VAR, with a summary heatmap of correlation coefficients. Fig. S15 Temporal variability of precipitation, snow depth, and air temperature at HYY and VAR. Notes S1 Thin sectioning of the tree rings. Notes S2 Isotope analysis for tree ring sections. Notes S3 Determination of the periods for each tree ring subsection. Notes S4 Pinitol correction on the WSCs. Notes S5 Correlations between pools and relative humidity and temporal inte [file NPH-247-97-s001.docx]

**New Phytologist Supporting Information**

**Opposing seasonal trends in source water and sugar dampen intra-annual variability in tree rings oxygen isotopes**

Paul Szejner ^1*^, Yu Tang ^1,2^, Charlotte Angove ^1^, Pauliina Schiestl-Aalto ^3^, Elina Sahlstedt ^1^, Giles Young ^1^, Nelson Daniel B. ^4^, Kahmen Ansgar ^4^, Matthias Saurer ^5^, Katja T. Rinne-Garmston ^1^.

**Article acceptance date: 18 April 2025**

**Figure S1.** Timing of water, wood, and sugar sample collection across individual trees at HYY.

**Figure S2**. Continuation. Timing of water, wood, and sugar sample collection across individual trees at HYY.

**Figure S3**. Continuation. Timing of water, wood, and sugar sample collection across individual trees at HYY.

**Figure S4**. Timing of water, wood, and sugar sample collection across individual trees at VAR.

**Figure S5**. Continuation. Timing of water, wood, and sugar sample collection across individual trees at VAR.

**Figure S6**. Continuation. Timing of water, wood, and sugar sample collection across individual trees at VAR.

**Figure S7**. Continuation. Timing of water, wood, and sugar sample collection across individual trees at VAR.

**Table S1**. Total number of samples collected per tree and soil depth at HYY.

**Table S2**. Total number of samples collected per tree and soil depth at VAR.

**Table S3**. Summary of collection dates, number of trees, and δ¹⁸O measurements for water, wood, and sugar pools at HYY.

**Table S4.** Summary of collection dates, number of trees, and δ¹⁸O measurements for water, wood, and sugar pools at VAR.

**Figure S8.** Distributions of δ¹⁸O values for water, sugar, and wood pools in HYY and VAR for 2018 and 2019.

**Figure S9.** Forest plot of pairwise group mean differences with 95 % confidence intervals from the Games-Howell test

**Figure S10.** Continuation. Forest plot of pairwise group mean differences with 95 % confidence intervals from the Games-Howell test

**Figure S11.** Forest plot of non-significant pairwise comparisons with 95 % confidence intervals from the Games-Howell test.

**Notes S1: Thin sectioning of the tree rings**

**Notes S2: Isotope analysis for tree ring sections**

**Notes S3: Determination of the periods for each tree ring subsection**

**Notes S4: Pinitol correction on the WSCs**

**Figure S12**. Comparison of δ¹⁸O in water-soluble carbohydrates before and after correction for pinitol influence.

**Notes S5: Correlations between pools and relative humidity and temporal integration periods**

**Figure S13**. Scatterplots of δ¹⁸O values from multiple pools versus relative humidity across 1–50 day integration windows at HYY, with a summary heatmap of correlation coefficients.

**Figure S14.** Scatterplots of δ¹⁸O values from multiple pools versus relative humidity across 1–50 day integration windows at VAR, with a summary heatmap of correlation coefficients.

**Figure S15**. Temporal variability of precipitation, snow depth, and air temperature at HYY and VAR.


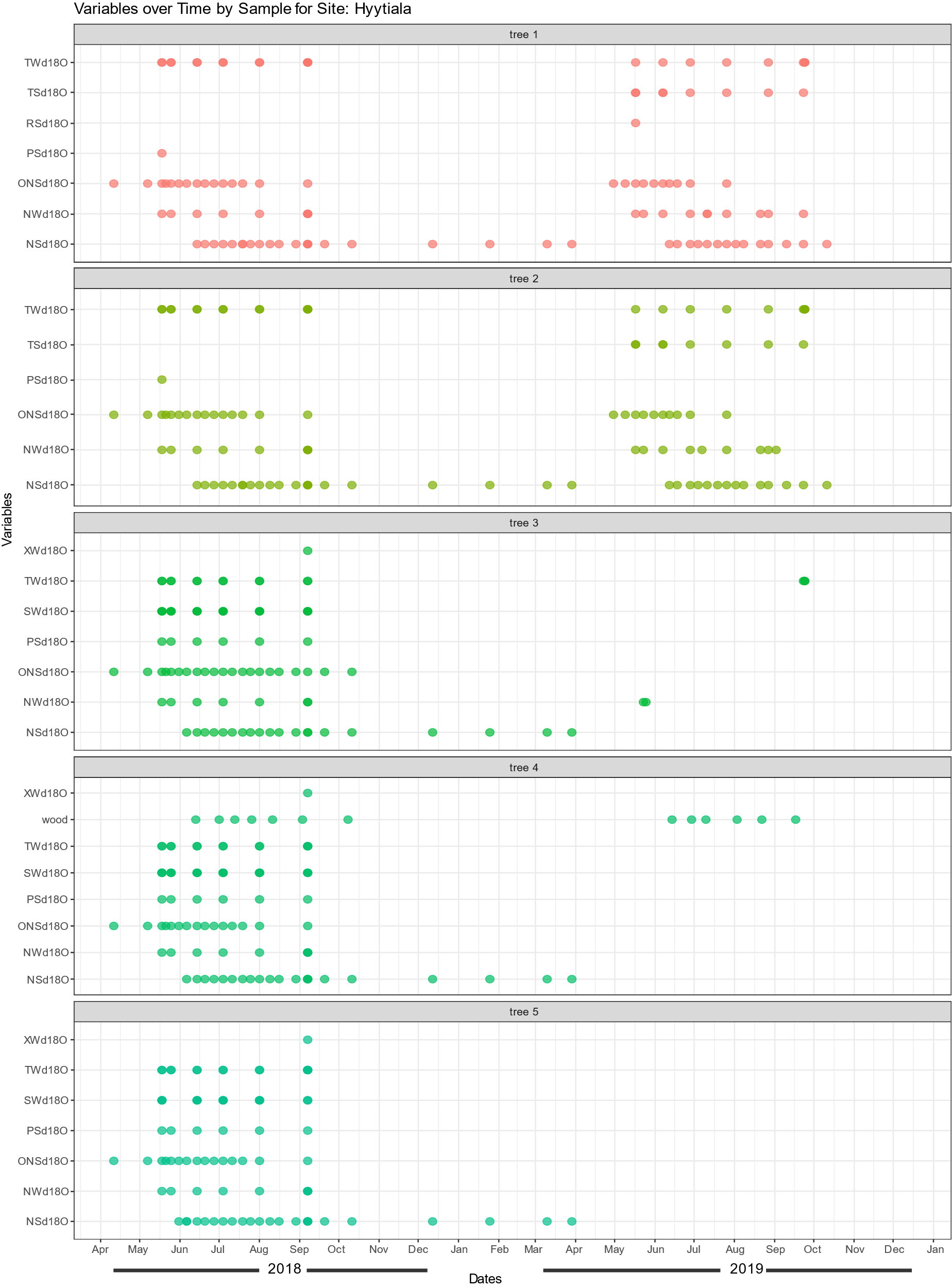


**Figure S1:** Shows the timing of sample collection across different trees over the 2-year sampling time **in HYY**. Each panel represents a specific tree, and within each panel, the dots indicate the times during the year when samples of water, wood, or sugars were taken.


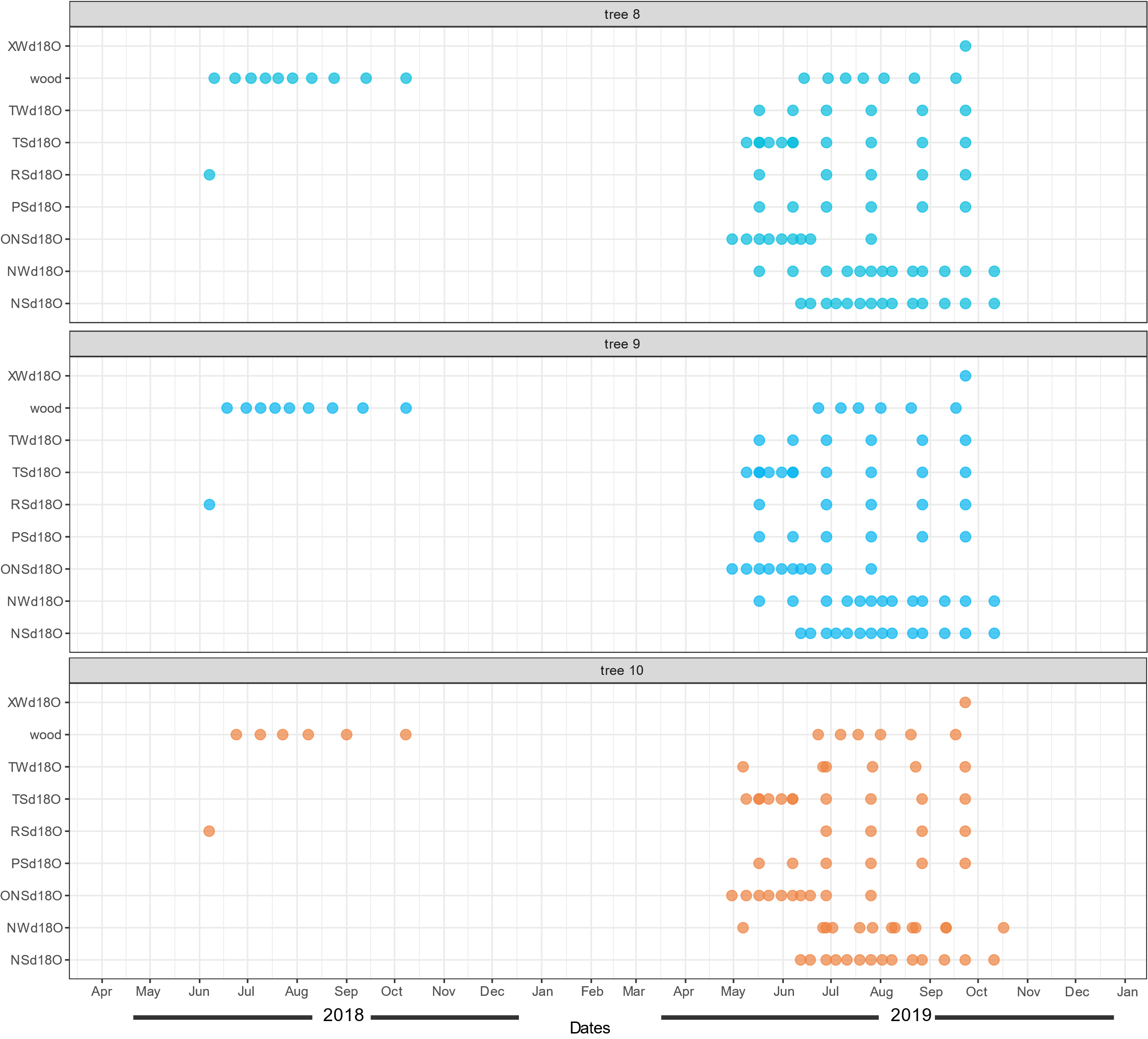


**Figure S2**, a continuation of figure S1, shows the timing of sample collection across different trees over the 2-year sampling time **in HYY**. Each panel represents a specific tree, and within each panel, the dots indicate the times during the year when samples of water, wood, or sugars were taken.


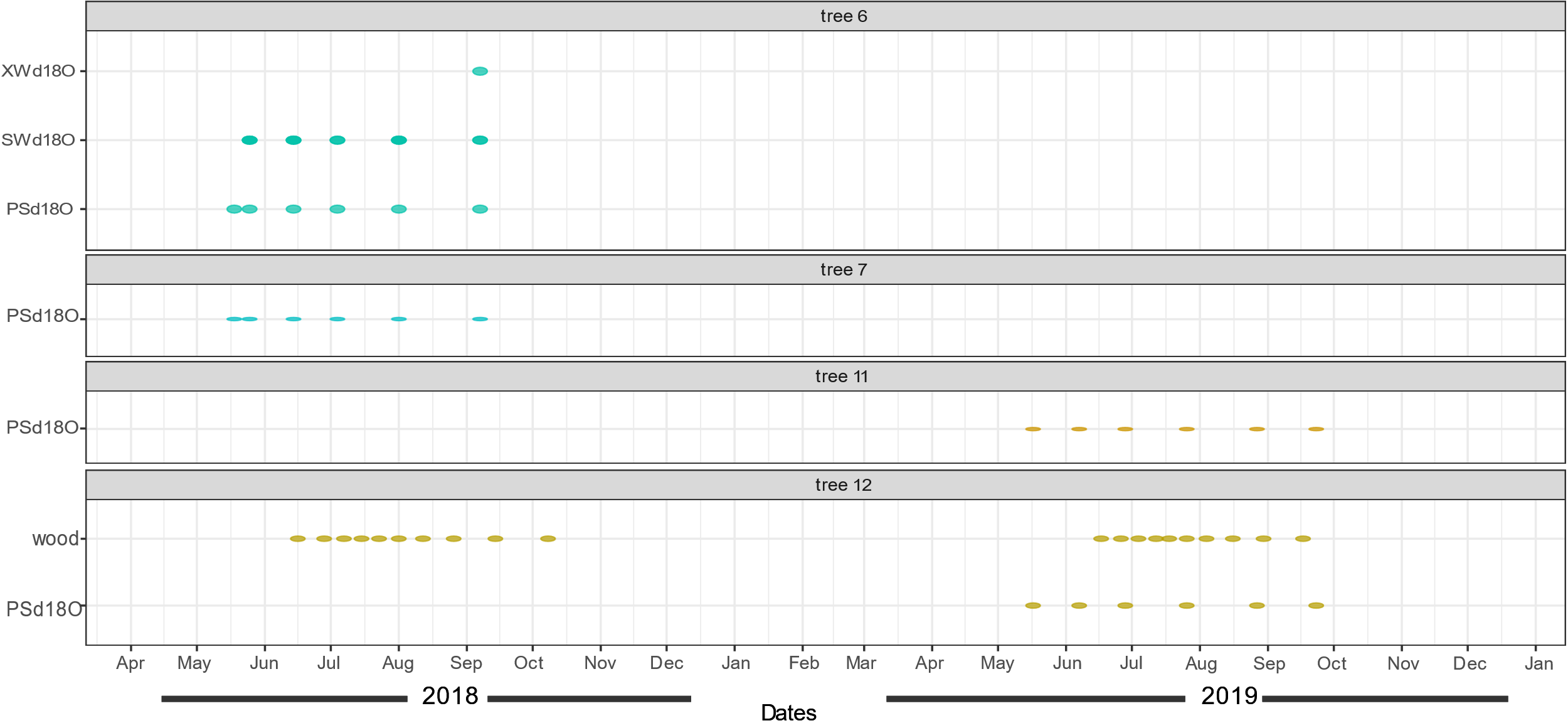


**Figure S3**, a continuation of figure S1,2, shows the timing of sample collection across different trees over the 2-year sampling time **in HYY**. Each panel represents a specific tree, and within each panel, the dots indicate the times during the year when samples of water, wood, or sugars were taken.


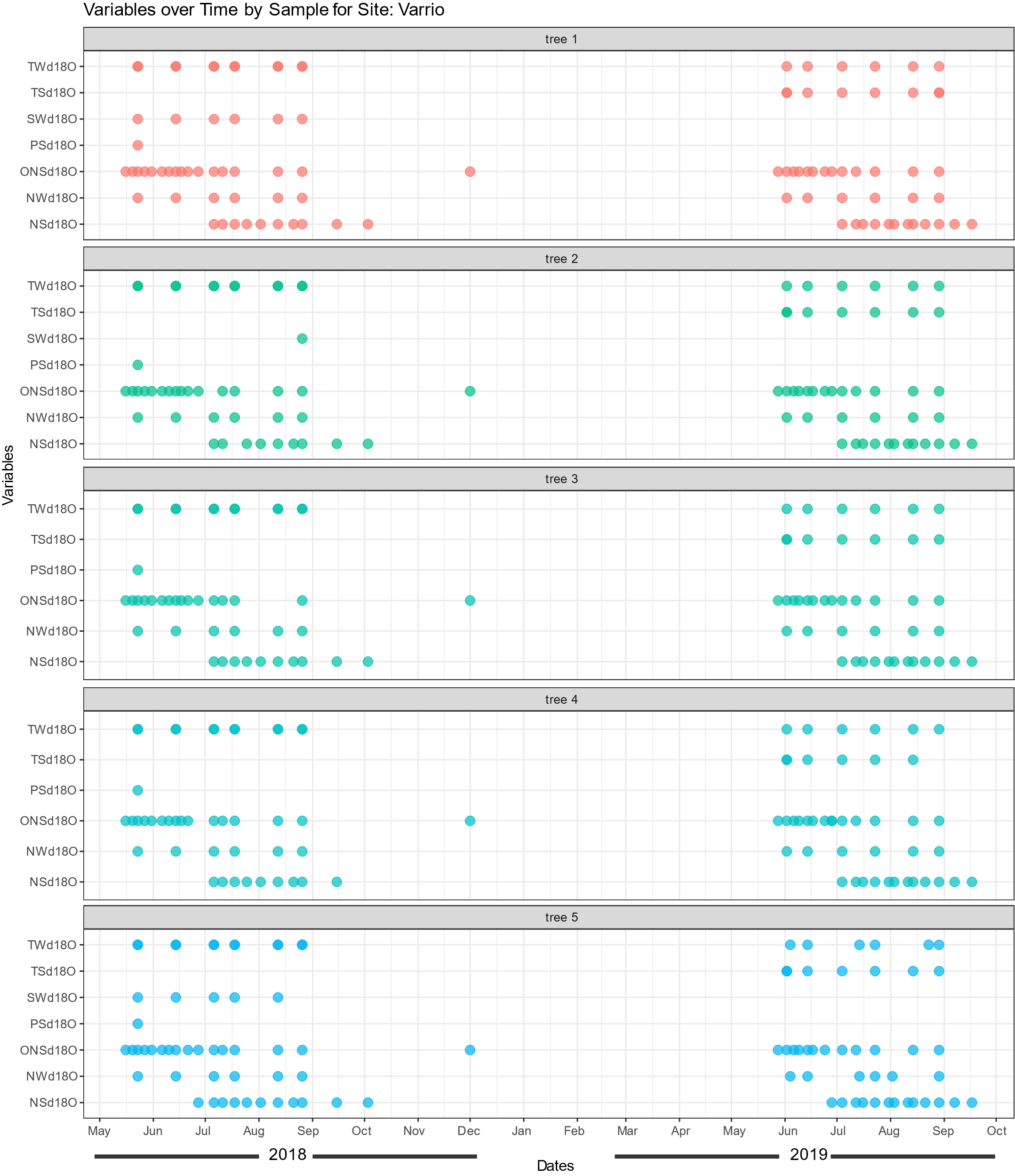


**Figure S4:** Shows the timing of sample collection across different trees over the 2-year sampling time **in VAR**. Each panel represents a specific tree, and within each panel, the dots indicate the times during the year when samples of water, wood, or sugars were taken.


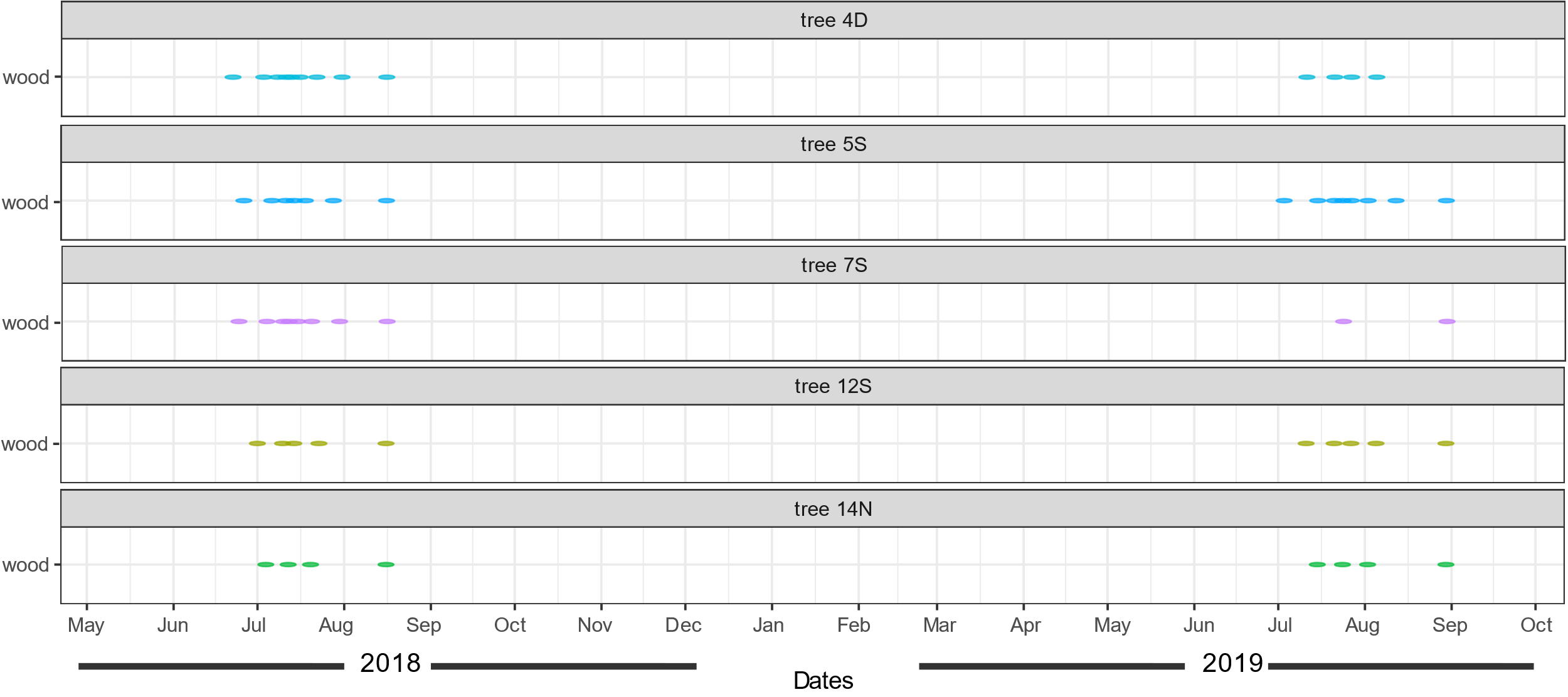


**Figure S5**: Shows the timing of sample collection across different trees over the 2-year sampling time **in VAR**. Each panel represents a specific tree, and within each panel, the dots indicate the times during the year when wood samples were estimated as the end of their formation and maturation process (figure 2).


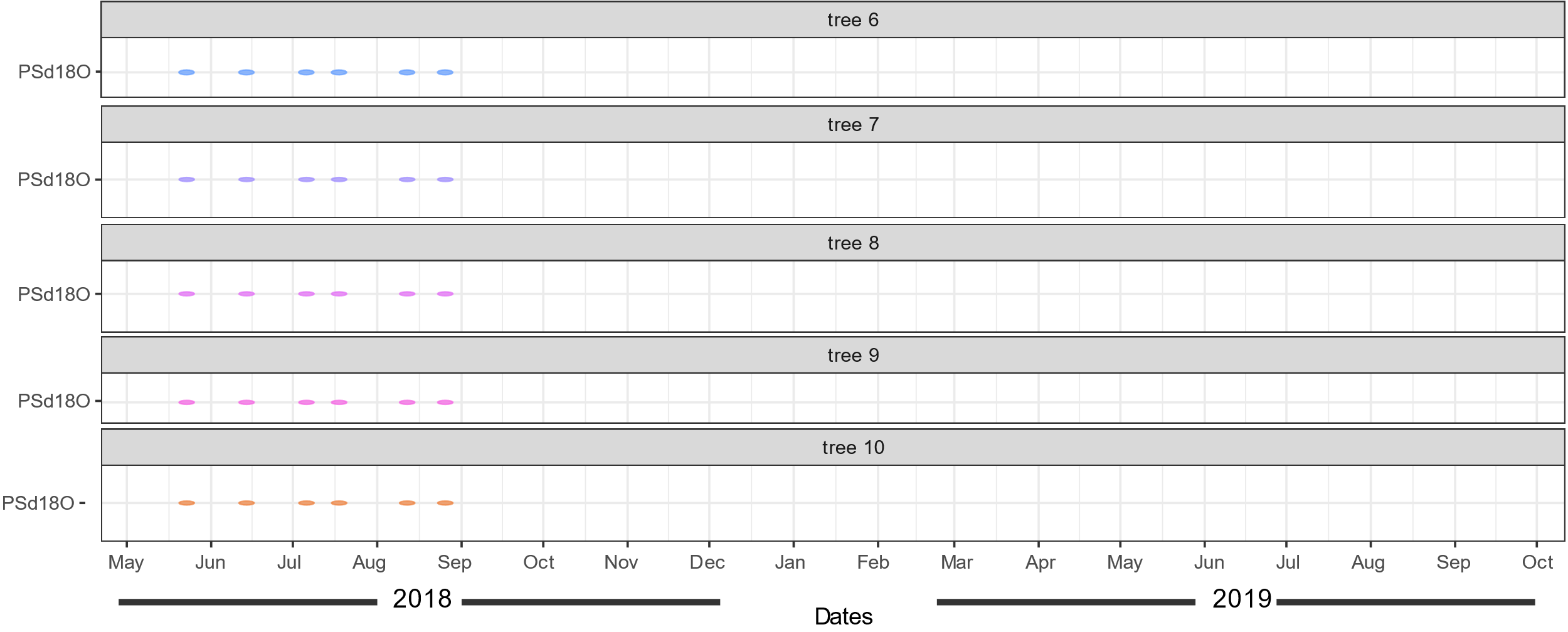


**Figure S6:** Shows the timing of sample collection across different trees over the 1-year sampling time **in VAR**. Each panel represents a specific tree, and within each panel, the dots indicate the times during the year when phloem sugars were taken.


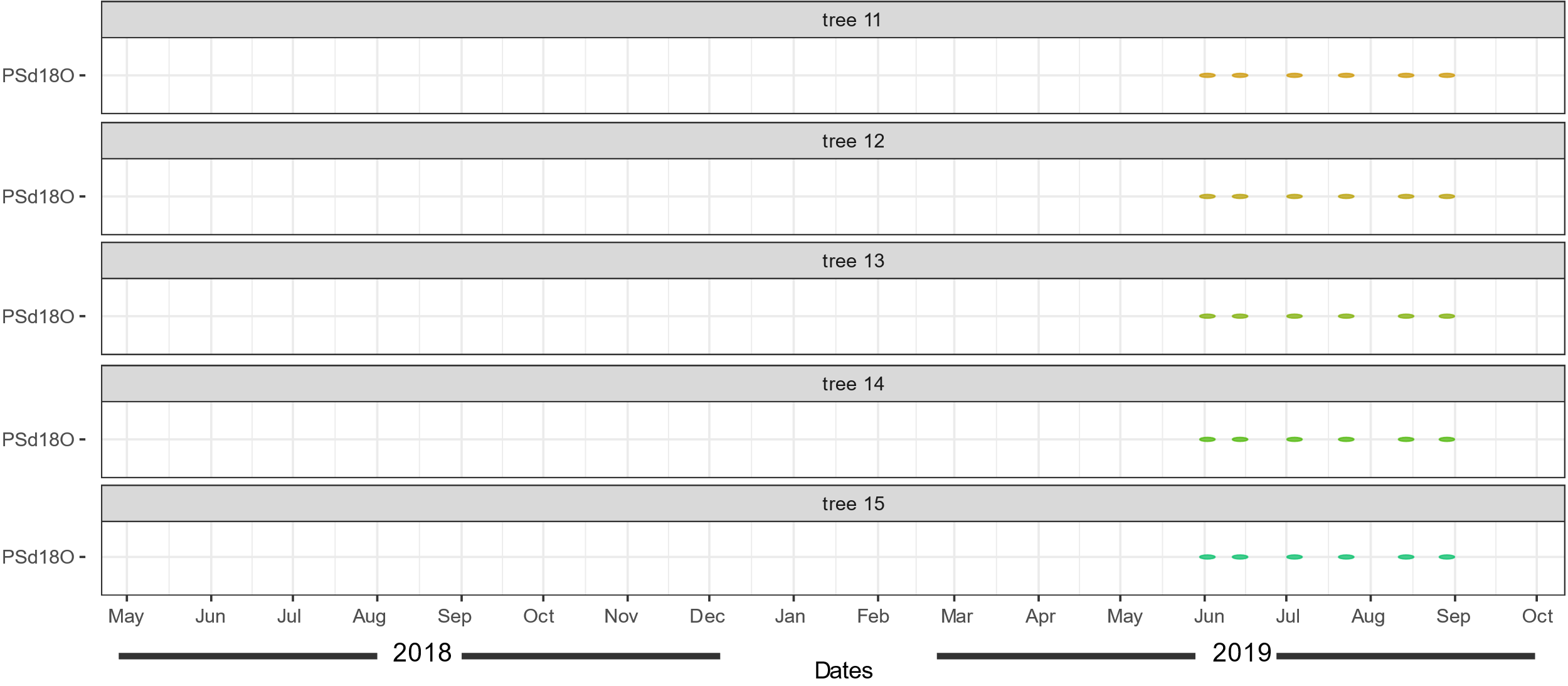


**Figure S7:** Shows the timing of sample collection across different trees over the 2-year sampling time in **VAR.** Each panel represents a specific tree, and within each panel, the dots indicate the times during the year when samples of water, wood, or sugars were taken.

**Table S1.** Total number of samples collected in HYY per tree and soil depth

**Table S2.** Total number of samples collected in VAR per tree and soil depth

**Table S3** Summary of data collection in HYY

| Variable | Collection dates | Number of trees | Number of d18O measurements |
| --- | --- | --- | --- |
| twig water (TWd18O) | 17 | 8 | 102 |
| xylem water (XWd18O) | 3 | 8 | 8 |
| needle water (NWd18O) | 31 | 8 | 98 |
| Wood (d18O) | 55 | 5 | 76 |
| root sugars ( RSd18O) | 6 | 4 | 18 |
| twig sugars (TSd18O) | 9 | 5 | 49 |
| Current year needle sugars ( NSd18O) | 34 | 8 | 175 |
| phloem sugars (PSd18O) | 12 | 12 | 62 |
| one-year-old needle sugars (ONSd18O) | 31 | 8 | 130 |

**Table S4** Summary of data collection in VAR

| Variable | Collection dates | Number of trees | Number of d18O measurements |
| --- | --- | --- | --- |
| twig water (TWd18O) | 15 | 5 | 90 |
| needle water (NWd18O) | 15 | 5 | 60 |
| Wood (d18O) | 33 | 5 | 56 |
| twig sugars (TSd18O) | 6 | 5 | 35 |
| phloem sugars (PSd18O) | 12 | 15 | 65 |
| Current year needle sugars (NSd18O) | 24 | 5 | 110 |
| one-year-old needle sugars (ONSd18O) | 30 | 5 | 146 |


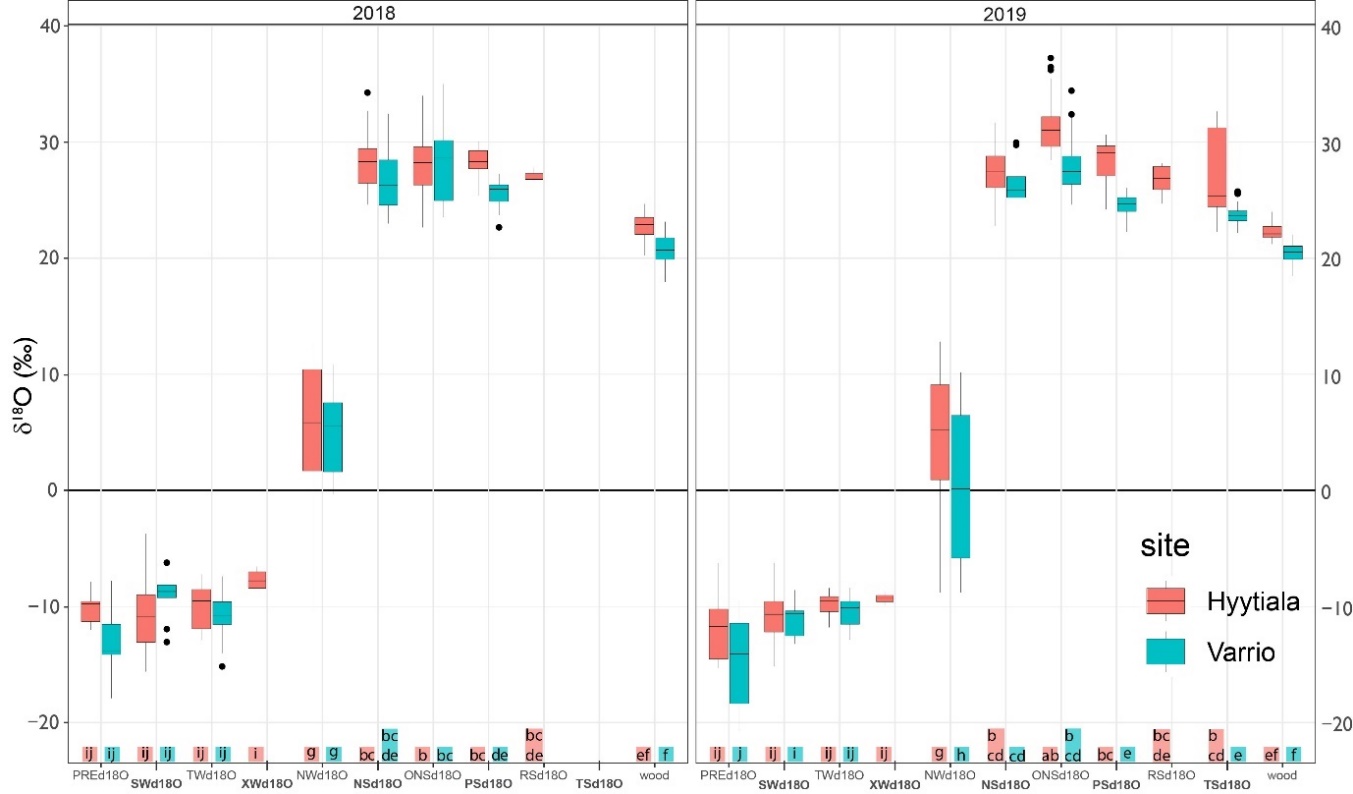


**Figure S8.** Isotopic distributions of various oxygen isotope variables for 2018 and 2019 studied in HYY and VAR. The x-axis denotes the main pools, while the y-axis represents the complete δ^18^O scale. The different colours indicate different sites. Based on Tukey's HSD test, groups with the same letters are not significantly different in their δ^18^O mean values—Boxplot based on grouped pools in Figure 3.


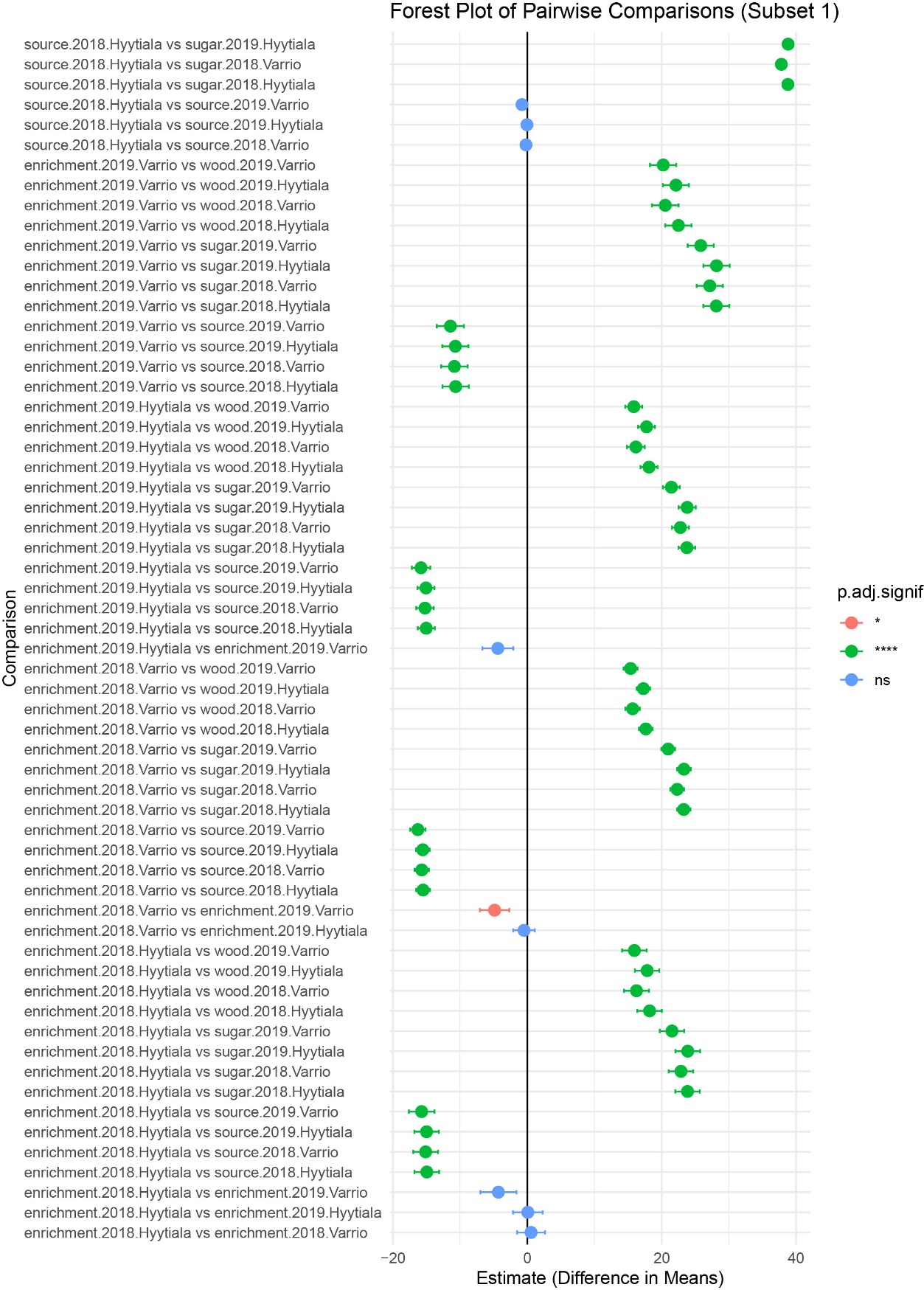


**Figure S9**. Forest plot of pairwise comparisons among groups using the Games-Howell test (Subset 1). The plot displays the estimated differences in group means (points) with their corresponding 95% confidence intervals (error bars) for all comparisons, arranged in alphabetical order. Significance levels are indicated by the adjusted p-value codes. (ns: Not significant (p > 0.05) *: p < 0.001 ****: p < 0.0001) In a few cases, minor rounding differences or adjustments for multiple comparisons may result in non-significant comparisons with confidence intervals that do not appear to cross zero.


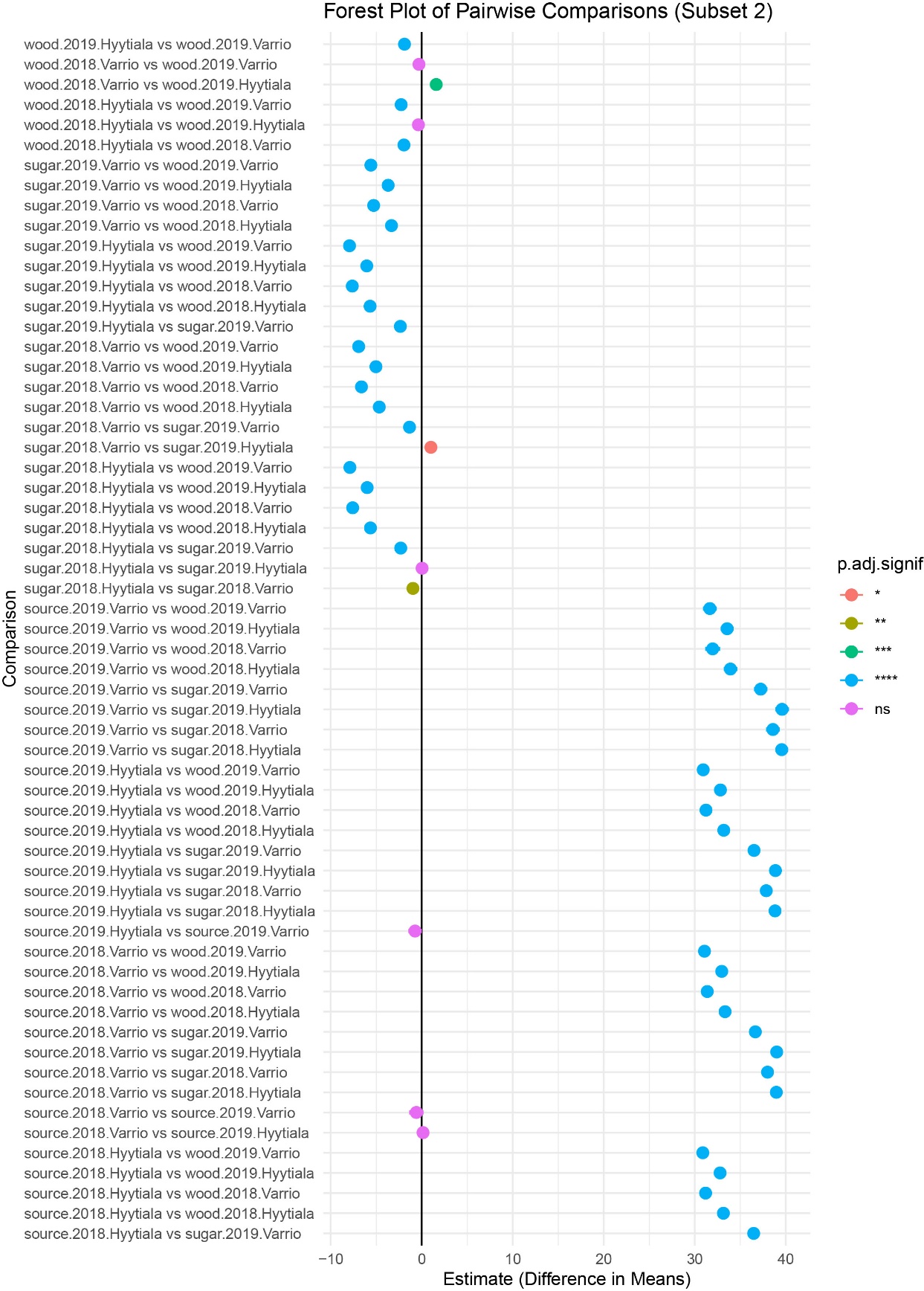


**Figure S10.** Forest plot of pairwise comparisons among groups using the Games-Howell test (Subset 2). As in Subset 1, the plot shows the estimated differences (points) and 95% confidence intervals (error bars) for each comparison, arranged alphabetically. The p-value significance codes (ns: Not significant (p > 0.05) *: p < 0.05 **: p < 0.01 ***: p < 0.001 ****: p < 0.0001) denote the levels of significance and note that slight discrepancies in the confidence interval display may occur due to rounding or multiple comparison adjustments.


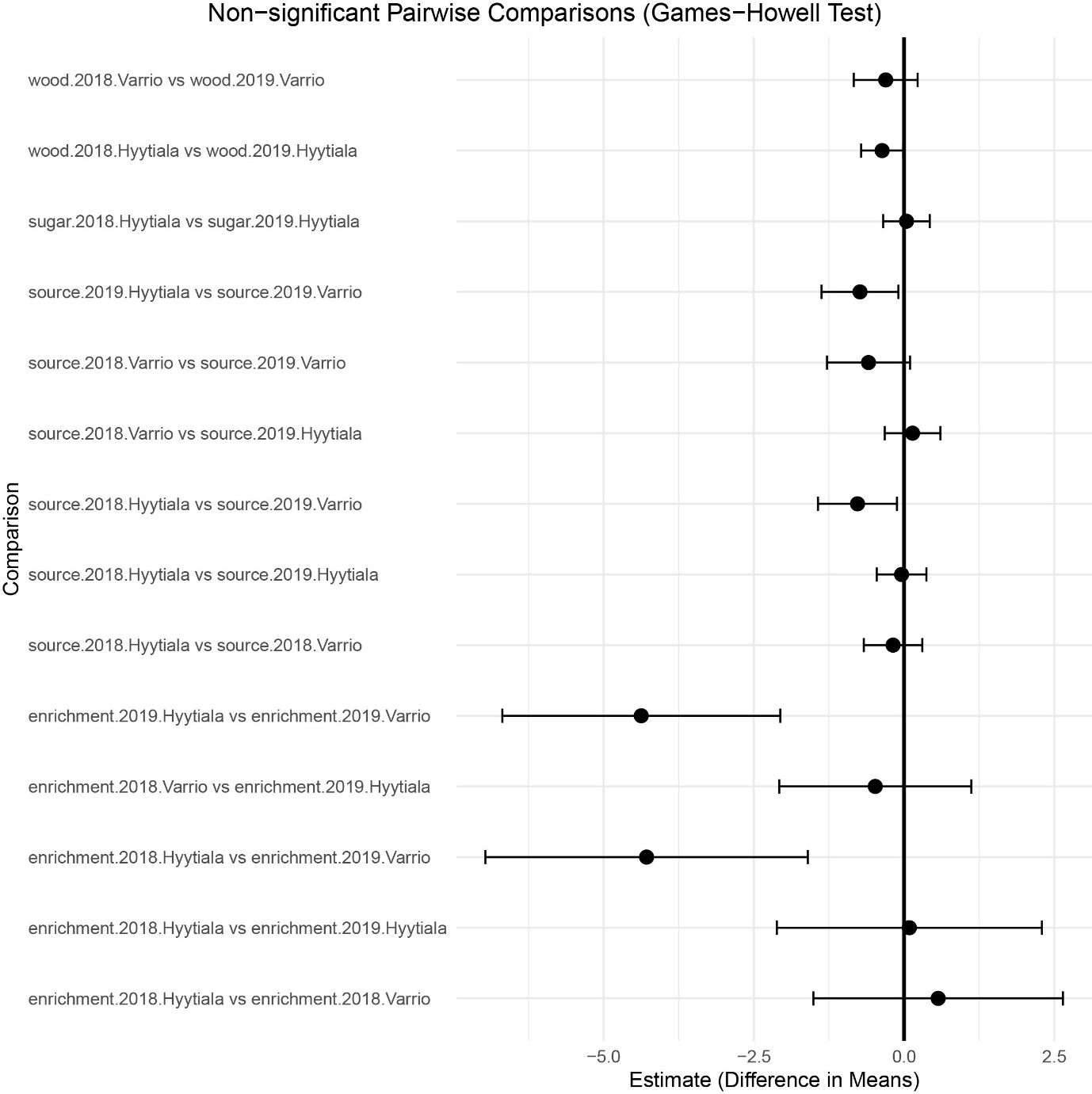


**Figure S11** Forest plot of non-significant pairwise comparisons among groups using the Games-Howell test. The plot displays the estimated differences in group means (points) along with their 95% confidence intervals (error bars). Comparisons are arranged in alphabetical order. Confidence intervals that include zero typically indicate no statistically significant difference between groups (p > 0.05). Note that in a few cases, minor rounding differences or adjustments for multiple comparisons may result in non-significant estimates with error bars that do not appear to cross zero.

# Notes S1: Thin sectioning of the tree rings

For stable oxygen isotope analysis, tree rings for 2018 and 2019, for five individual trees from each site, were divided into thin sections (c. 80µm) using a cryo-microtome (Leica CM3050S). Cores were mounted vertically on the cryo-microtome sectioning plate using frozen distilled water, and a flexible industrial endoscope was used to aid in precision cutting. As the rings of the individual trees varied in width, the number of thin sections from each tree varied considerably (Figure 2). For example, only two sections could be collected for the narrowest ring instead of larger rings, where as many as 19 sections were obtainable for an individual year. Because of this significant disparity in sample numbers per ring, it was decided to reduce the number of analyses for the rings with large numbers of sections (≥14); in these instances, only the odd-numbered sections were analysed (counting from the bark side inwards). This produced a reasonable seasonal isotope profile while significantly reducing the number of individual analyses required.

# Notes S2: Isotope analysis for tree ring sections

The wood sample δ^18^O analyses were performed using a Flash IRMS elemental analyser operated in pyrolysis mode with a glassy carbon reactor operated at 1400 degrees coupled to a Delta V Plus isotope ratio mass spectrometer (IRMS) through a ConFlo IV interface, controlled by a computer running the Isodat 3.0 software platform (Thermo Fisher Scientific, Bremen, Germany). Samples were analysed using a Costech Zero-Blank Autosampler (NC Technologies Srl, Milan, Italy), which was sealed and purged with helium with the samples inside and then opened to the pyrolysis reactor and left to equilibrate for at least three hours prior to analysis. Instrument values were normalized to the VSMOW/SLAP scale using calibrated in-house standards with δ^18^O values of +2.89 ‰, +8.91 ‰, and +23.96 ‰. These standards were a dimethyl benzoic acid, a trimethyl benzoic acid, and a benzoic acid. Separate aliquots of the trimethyl benzoic acid were used to perform scale normalization and to serve as QC samples. Additional QC samples were sometimes used. This is often commercial cellulose and an in-house prepared bulk leaf sample. Measurement accuracy was calculated as the offset between the mean measured value of the trimba QC and the calibrated value of +8.91 ‰ (offset (i.e., epsilon) = alpha-1 = (dmeasured+1/dcalibrated+1)-1.). The long-term analytical precision in the lab is calculated as the standard deviation of the measured trimba QC δ^18^O values from 2016-07-11 to 2023-03-20 and is 0.2 ‰ (n = 677). Long term precision from 2022-01-01 to 2023-03-20 is 0.2 ‰ (n = 88).

# Notes S3: Determination of the periods for each tree ring subsection

Micro-cores with a diameter of 2 mm and length of 15 mm were collected at the height of 1.3 m from five mature trees using a Trephor corer (Costruzioni Meccaniche Carabin C., Belluno, Italy) (Rossi *et al.*, 2006). The xylogenesis observations of the pheno-dates (Tang et al. 2022) were used to determine the yearly tree-ring growth curve for each site, which were monitored through two methods: xylogenesis observations for the years 2018-2019 (Tang *et al.*, 2023) and the dynamic growth model Carbon Allocation Sink Source Interaction (CASSIA, Schiestl-Aalto, et al. 2015). Micro-core sections were prepared and analysed (Jyske et al., 2014) to establish the timing and duration of current-year tracheids from their formation to the end of the maturation phases (Morino *et al.*, 2021). The growth curves for tracheid production and maturation were determined using Gompertz fitting. In contrast, the dimensional growth curve of tracheid production and the number-based growth curve of tracheid maturation were modelled with CASSIA (Schiestl‐Aalto *et al.*, 2015). The number-based growth curves were converted to dimensional growth curves via a non-linear fitting curve. By knowing the growth curves and the relative position of each δ^18^O subsection of the ring, the growth period for each measurement was determined, which was then used for timing intra-seasonal tree-ring δ^18^O. The R code for this analysis is available.

# Notes S4: Pinitol correction on the *WSCs*

We corrected the bulk sugars to reduce isotopic influence by sugar alcohols like pinitol, which has a relatively constant δ^18^O of 25‰. (Leppä *et al.*, 2022). To achieve this, assuming extracted and purified WSCs are a mixture of sugars and sugar alcohols, we used Eqn 7 from Leppä et al. (2022):

R_WSCs_= (S_s_ * R_s_ + S_p_ * R_p_) / (S_s_ + S_p_)

In this equation:

• R_WSCs_ is the isotopic ratio of needle water-soluble carbohydrates (*WSCs*).

• S_s_ represents the concentration of sugars (sucrose + hexoses).

• R_s_ is the isotopic ratio of sugars (sucrose + hexoses), the variable we aim to solve for.

• S_p_ denotes the concentration of sugar alcohols (e.g., pinitol).

• R_p_ is the isotopic ratio of sugar alcohols (e.g., pinitol).

To solve for R_s_, we rearranged the formula:

R_s_ = [(R_WSCs_* (S_s_ + S_p_)) - (S_p_ * R_p_)] / S_s_

The following data is estimated based on measurements in the same site to calculate Rs. Where generally was found % of pinitol in needles, phloem, and twigs.

By applying this correction, we can obtain the adjusted isotopic ratio of sugars (R_s_), correcting the influence of sugar alcohols, like pinitol, and ensuring a more accurate representation of the isotopic signature of sugars in the sample.


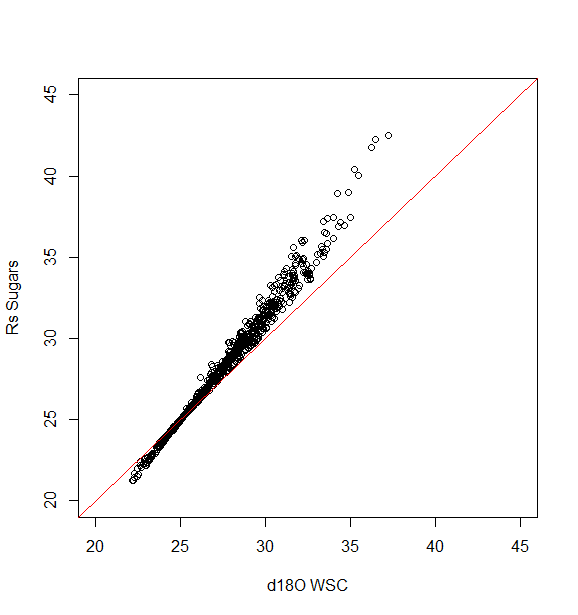


**Figure S12.** Comparison between δ^18^O of WSCs and the values corrected for sugars without the influence of pinitol. 2019 values are estimated using the **mean pinitol concentrations** measured in 2018, **Which tend to be 28.6 ug/mg in most samples**. The 95% confidence interval from the measurements was [28.04 ug/mg -- 29.16 ug/mg], and no correlation between the δ^18^O composition and concentration was found r = 0.05, so it is reasonable to use this assumption to correct the 2019 values that we don’t have specific concentrations per sample.

It is worth noting that Angove et al. (2025) found some differences in pinitol concentrations and relative abundance through the season. Therefore, when extrapolating this correction to some observations lacking compound-specific analysis, there is a possibility of inaccuracy. Nonetheless, we assume that this estimation at least partially accounts for the pinitol influence.

# Notes S5: Correlations between pools and relative humidity and temporal integration periods

A hypothesis-driven approach was implemented to better understand the relationships between the different δ^18^O pools and environmental variables. The hypothesis centres around the temporal integration of the effects of RH over various time scales, starting at 1, 5, 10, 15, 20, and 25 days, and their correlation with the different δ^18^O pools.

One aspect of this hypothesis seeks to assess if the needle water δ^18^O is more tightly coupled to a shorter integration period of RH, as explored in Leppä et al. (2022). This is based on the premise that needle water δ^18^O reflects more recent environmental conditions due to the relatively quick uptake and transpiration processes. Furthermore, the δ^18^O values of sugars, which reflect isotopic signatures accumulated during the photosynthetic assimilation process, potentially correlate with an extended integration period. This integration suggests that the measured photoassimilates might represent a blend of sugars, including those synthesized recently and lingering sugars from previous days, spanning approximately 2-5 days (Leppä *et al.*, 2022; Tang *et al.*, 2022). The δ^18^O values in resin-extracted wood, as analysed in (Tang *et al.*, 2022), indicate a prolonged integration period, likely encompassing about a month—the slow growth of trees and mixed sugar isotopic signals in the phloem result in an extended timeframe. Thin sections can offer a month-long snapshot of environmental influences on the tree. This approach aims to determine the most suitable period of integration that accurately reflects the drivers that govern interactions between oxygen isotopes, sources, and relative humidity at these sites.


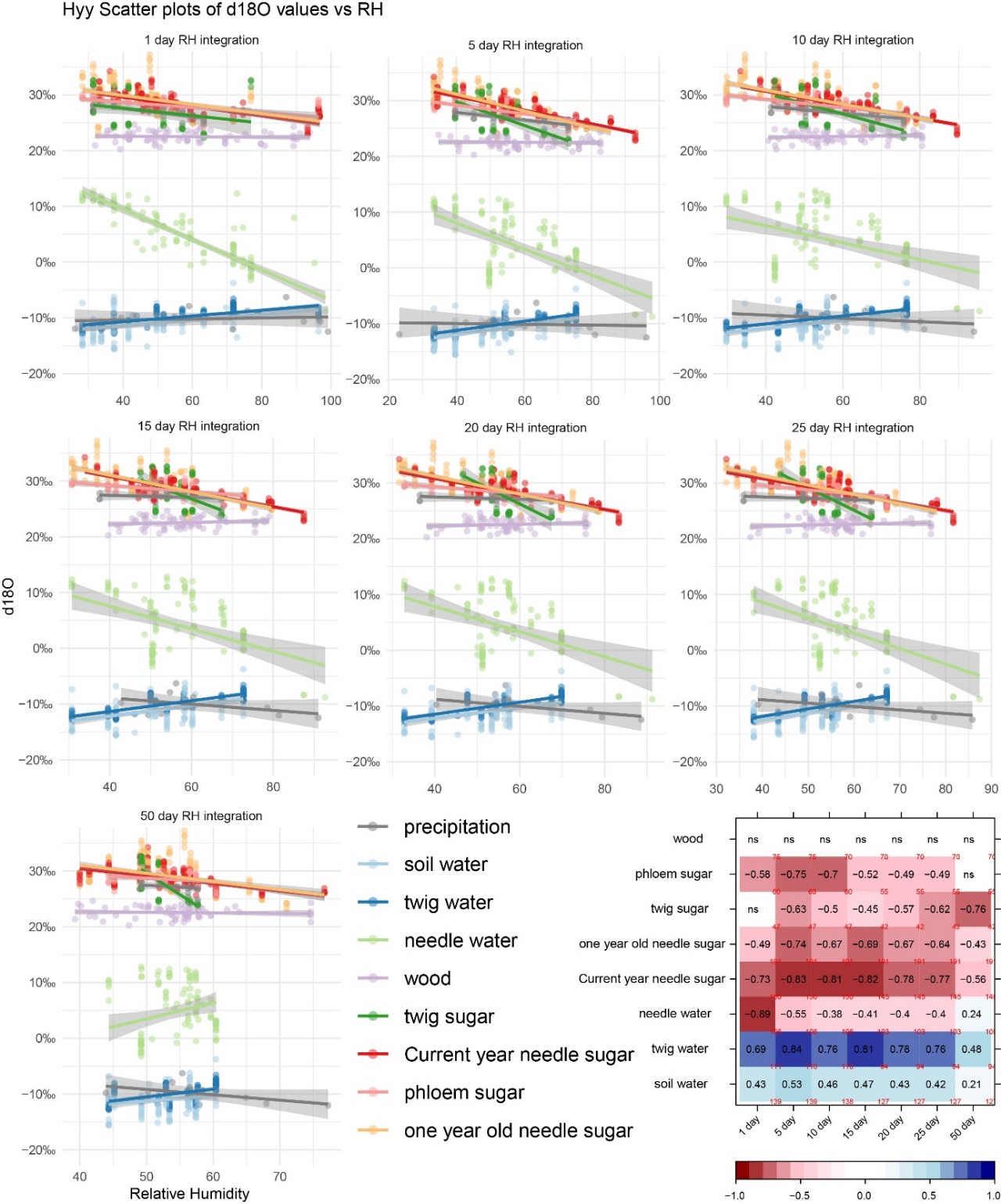


**Figure S13:** Scatterplots illustrating the relationship between δ¹⁸O values from various water, sugar, and wood pools against relative humidity (RH) across different temporal integration windows, ranging from 1-day to 50-day averages in HYY. Each scatterplot represents a specific RH integration period (1-day, 5-day, 10-day, 15-day, 20-day, 25-day, and 50-day), with δ¹⁸O values plotted on the y-axis and RH on the x-axis. The color-coded lines correspond to different pools: precipitation (gray), twig water (blue), soil water (purple), needle water (green), wood (dark green), twig sugar (orange), phloem sugar (yellow), current year needle sugar (red), and one-year-old needle sugar (pink). The bottom-right heatmap summarizes the correlation coefficients for each relationship, with darker shades of red indicating stronger negative correlations and blue for the positive correlations and non-significant (ns) correlations.


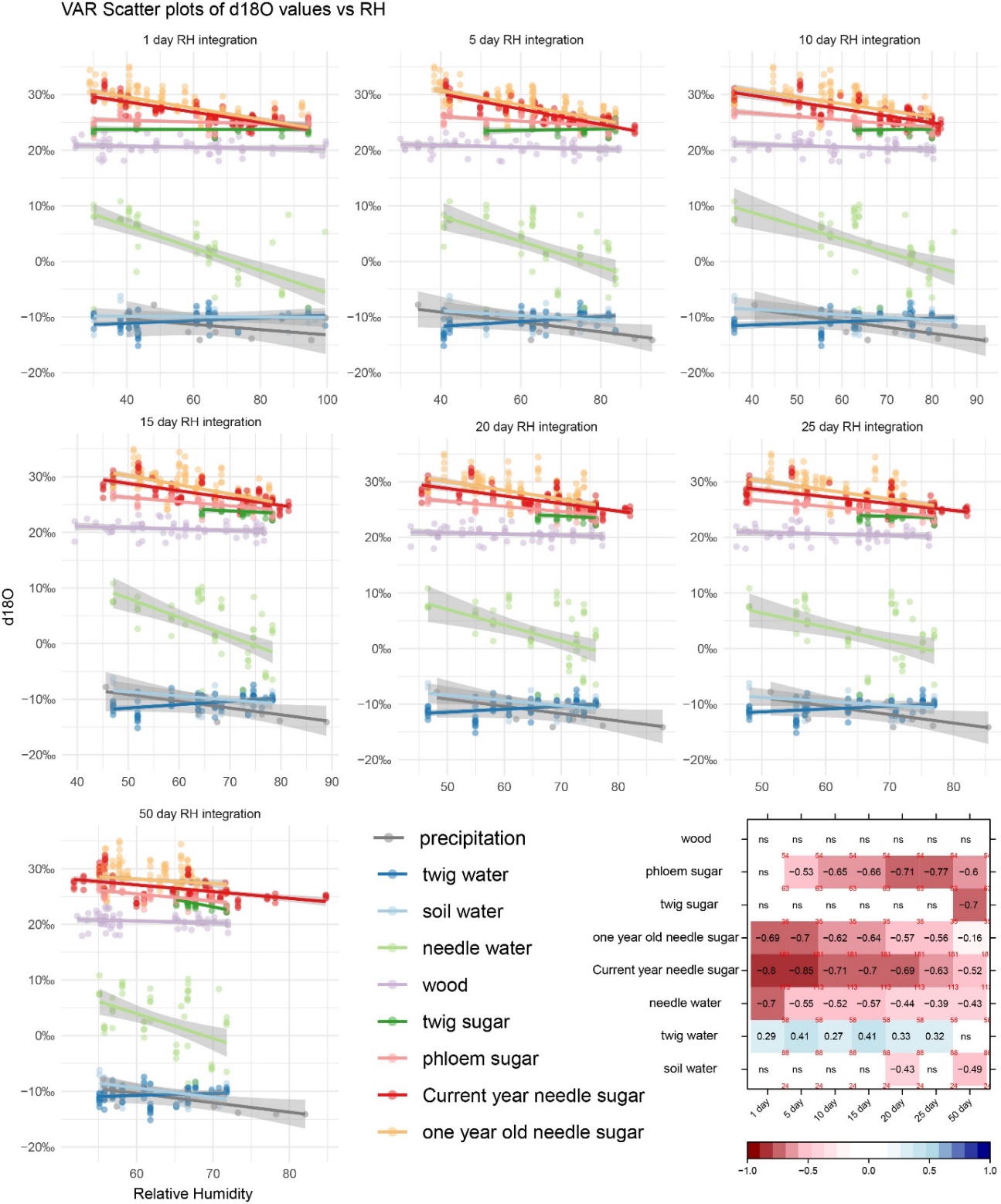


**Figure S14:** Scatterplots illustrating the relationship between δ¹⁸O values from various water, sugar, and wood pools against relative humidity (RH) across different temporal integration windows, ranging from 1-day to 50-day averages in VAR. Each scatterplot represents a specific RH integration period (1-day, 5-day, 10-day, 15-day, 20-day, 25-day, and 50-day), with δ¹⁸O values plotted on the y-axis and RH on the x-axis. The color-coded lines correspond to different pools: precipitation (gray), twig water (blue), soil water (purple), needle water (green), wood (dark green), twig sugar (orange), phloem sugar (yellow), current year needle sugar (red), and one-year-old needle sugar (pink). The bottom-right heatmap summarizes the correlation coefficients for each relationship, with darker shades of red indicating stronger negative correlations and blue for the positive correlations and non-significant (ns) correlations.


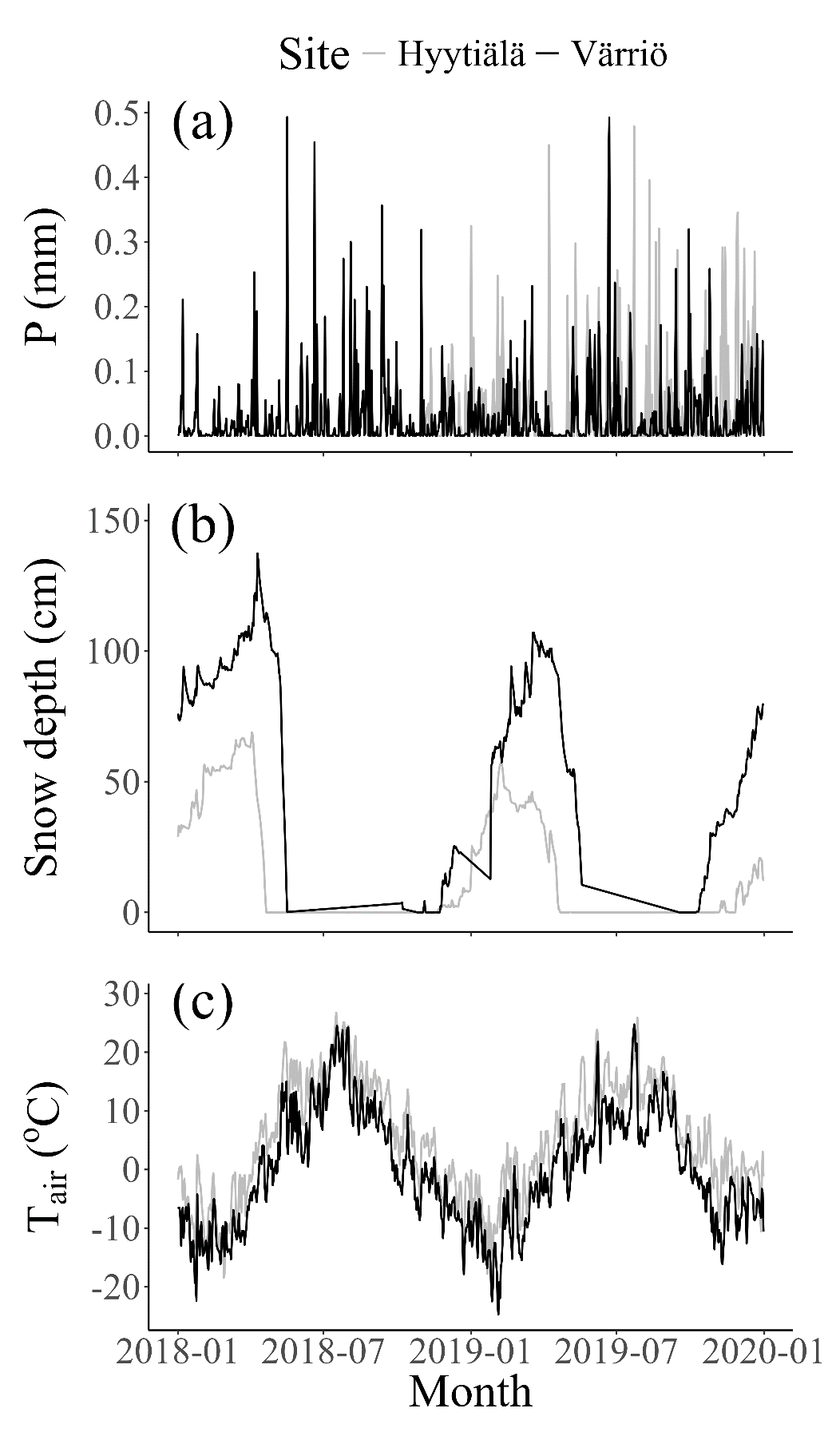


**Figure S15** Temporal variability of meteorological parameters at Hyytiälä and Värriö. (a) Precipitation (P), (b) snow depth, and (c) air temperature (T_air_) Data sources are ICOS and SMEAR..

1. References:

**Angove C, Wiesenberg GLB, Lehmann MM, Saurer M, Tang Y, Sahlstedt E, Speckert TC, Schiestl‐Aalto PP, Rinne‐Garmston KT**. **2025**. Time‐integrated δ^2^ H in *n* ‐alkanes and carbohydrates from boreal needles reveal intra‐annual physiological and environmental signals. *New Phytologist* **246**: 498–514.

**Leppä K, Tang Y, Ogée J, Launiainen S, Kahmen A, Kolari P, Sahlstedt E, Saurer M, Schiestl‐Aalto P, Rinne‐Garmston KT**. **2022**. Explicitly accounting for needle sugar pool size crucial for predicting intra‐seasonal dynamics of needle carbohydrates δ ^18^ O and δ ^13^ C. *New Phytologist* **236**: 2044–2060.

**Morino K, Minor RL, Barron-Gafford GA, Brown PM, Hughes MK**. **2021**. Bimodal cambial activity and false-ring formation in conifers under a monsoon climate. *Tree Physiology*: 1–13.

**Rossi S, Anfodillo T, Menardi R**. **2006**. Trephor: A New Tool for Sampling Microcores from tree stems. *IAWA Journal* **27**: 89–97.

**Schiestl‐Aalto P, Kulmala L, Mäkinen H, Nikinmaa E, Mäkelä A**. **2015**. CASSIA – a dynamic model for predicting intra‐annual sink demand and interannual growth variation in S cots pine. *New Phytologist* **206**: 647–659.

**Tang Y, Schiestl-Aalto P, Lehmann MM, Saurer M, Sahlstedt E, Kolari P, Leppä K, Bäck J, Rinne-Garmston KT**. **2023**. Estimating intra-seasonal photosynthetic discrimination and water use efficiency using δ13C of leaf sucrose in Scots pine (J Lunn, Ed.). *Journal of Experimental Botany* **74**: 321–335.

**Tang Y, Schiestl-Aalto P, Saurer M, Sahlstedt E, Kulmala L, Kolari P, Ryhti K, Salmon Y, Jyske T, Ding Y, *et al.*** **2022**. Tree organ growth and carbon allocation dynamics impact the magnitude and δ13C signal of stem and soil CO2 fluxes (L Cernusak, Ed.). *Tree Physiology* **42**: 2404–2418.
